# Patterns between evidence seeking behaviors, reasoning, and cognitive reflection: A supervised clustering approach

**DOI:** 10.1371/journal.pone.0352096

**Published:** 2026-06-25

**Authors:** Chénangnon Frédéric Tovissodé, Florian Justwan, Bert Baumgaertner

**Affiliations:** 1 Institute for Modeling Collaboration and Innovation, University of Idaho, Moscow, Idaho, United States of America; 2 Department of Politics and Philosophy, University of Idaho, Moscow, Idaho, United States of America; University of Granada: Universidad de Granada, SPAIN

## Abstract

In the face of risk, decision-making can be driven by styles of evidence gathering and information processing. When studying how people navigate the complex landscapes of evidence, researchers face the analytical problem of an exponentially growing number of distinct evidence gathering styles, as the number of pieces of information increases. The existing solution is to chunk information into a manageable number of pre-defined categories. In this work, we propose to meet this analytical challenge with a two-pronged strategy. First, our observational setting offers more fine-grained pieces of evidence but masks the content of evidence behind a query (e.g., ’what does so-and-so say?’) to ensure that people only access what they deem as potentially relevant. Second, applying supervised clustering based on the SHapley Additive exPlanation (SHAP) methodology allows us to relate evidence from gathered patterns to final decision while significantly relaxing theoretical delineations of evidence types that would otherwise make analysis intractable. We argue that this two-pronged strategy approximately integrates the pathway from evidence seeking to information processing and decision-making. We applied this strategy to demonstrate the fruitfulness of bridging work on evidence gathering and information processing. For example, one mental shortcut (heuristic) to arrive at decisions when assessing a causal claim using a 2×2 contingency table is the ‘base rate neglect heuristic’ (considering only the treatment group, comparing the number of positive outcomes to the number of negative outcomes). While base rate neglect is a well-established heuristic in information processing research, there is not yet a clear equivalent picture regarding evidence gathering. We develop this picture by considering the assessment of the effectiveness of a hypothetical nasal spray based on queries that span gathering/processing evidence categories. Using a demographically diverse online sample collected during August 2024 in the United States, we establish that the base rate neglect heuristic from information *processing* research is also a heuristic when it comes to *gathering* first-order data. For example, we find that higher performance on the cognitive reflection test predicts selection of the full data of a 2×2 contingency table. But, the latter group is nevertheless similar to base rate neglecters in who they consider to be relevant outside sources (i.e., they have roughly the same “deference” behavior). So while evidential categories in information *seeking* research are well suited to track differences in deference behaviors, they are blind to the difference between these two groups. These findings are additionally important for designing tailored health communication so as to avoid fallacious inferences.

## Introduction

Understanding how people navigate landscapes of evidence is fundamental to fostering trust in science [1,2], strengthening democratic discourse [3], and even improving public health outcomes [4]. This understanding is challenged by complex feedbacks between how a person gathers evidence and reasons from it – having processed information from one source can change what sources a person gathers from next and how they interpret new information [5–7]. This complexity is particularly high in the early stages of emerging information about the causal efficacy of an intervention, i.e., before bodies of evidence converge. During periods of the COVID-19 pandemic, for instance, people were forced to navigate an “infodemic” of conflicting claims about the efficacy of interventions such as imposed social distancing, face-mask wearing, and vaccination.

Researchers who study how people navigate this complex landscape face their own kind of problem. Without chunking information into a manageable number of categories, the number of distinct combinations of pieces of information for consideration grows exponentially; with five pieces of information there are 2^5^ = 32 distinct patterns, with ten it is 2^10^ = 1024. To make this tractable, researchers group pieces of information into a typology of evidence types, e.g., “statistical”, “scientific”, “expert testimony”, etc. (more below), allowing them to study relationships between *the categories* and other variables of interest. A problem emerges, however. As the next section illustrates, there are many plausible evidence typologies that chunk information in different, even incompatible ways. This presents a challenge for how to synthesize insights across typologies. Our primary contribution in this paper is to illustrate an approach that can help address this problem and better facilitate interdisciplinary insights along the full pathway from evidence gathering, information processing, and decision making.

### A tangled mess of evidence types

The complex interplay between evidence gathering and information processing tends to force us to hold fixed (or ignore) some parts of the pathway in order to manipulate other parts. For example, by controlling what information subjects are *given*, we can isolate pre-defined (social) psychological variables that impact how the information is *processed*, as exemplified by results in motivated reasoning [8–11] or cultural cognition [12,13].

Alternatively, to gain insights about information *seeking* behaviors, the reigns of selection are handed over to subjects, but constrained by pre-defined typologies of evidence. For example, studies using the Risk Information Seeking and Processing (RISP) model generally distinguish between scientific, statistical, experiential, and expert evidence [14–16], while other theoretical delineations include anecdotal, statistical, causal, and expert evidence [17], or just anecdotal versus scientific evidence [18,19]. How these types are defined varies and come with tradeoffs. We will briefly illustrate those that are salient for our purposes.

First, consider “statistical” evidence. Consider, for example, using a 2×2 contingency table to evaluate the effectiveness of face-mask wearing during the first COVID-19 wave (the number of individuals who wore or did not wear face-mask, and got or did not get sick). People who consider only one cell value (e.g., the number of individuals who wore face-mask and got sick) to assess the link between face-mask wearing and COVID-19 infection are using *categorical* evidence. By contrast, *associative* evidence is a set of two or more aggregates (categories) of data points that are compared to support or oppose, e.g., a causal claim. And depending on what information in a 2×2 table is being considered, associative evidence can be *partial* (e.g., comparing the number of individuals who wore face-mask and got sick to the number of individuals who did not wear face-mask and got sick) or *full* (e.g., comparing the proportion of people who got sick among those who wore face-masks to the proportion of people who got sick among those who did not wear face-masks). These fine-grained distinctions of “statistical data” are crucial. For example, work on motivated numeracy shows that cognitive heuristics can (fallaciously) lead to dramatically different conclusions than more “complete” interpretations, and can depend on whether the issue is related to, e.g., ideological beliefs [9] (more below). Recent work echoes these dependencies in the context of understanding information seeking as well [20]. “Statistical” may be too monolithic of a category.

Second, consider “expert” evidence. At first glance this seems intuitive enough: people rely on an external authority such as a public health institution, regulatory body, or clinical expert, to make decisions about matters they know less about. But upon reflection, this type of evidence is quite nuanced. What makes up expertise depends itself on complex considerations. Here it is helpful to acknowledge a distinction between first-order evidence (i.e., information that directly supports the truth or falsity of a claim) and higher-order evidence (i.e., information that supports the relevance of a source) [21,22]. Part of the complication is that some data can be first-order in one context, e.g., the weather forecast on a particular day, or higher-order evidence in another, e.g., as part of the history of forecasts to assess a source’s reliability. Interpretations of the same data as first-order or higher-order evidence have been documented in the information processing and gathering literature [20,23,24]. Moreover, people have sophisticated strategies for how they weight and assign expertise in relation to how they might independently process first-order data [25].

Understandably then, those focused on information processing tend to omit the role of outside sources. But that generates a gap in our understanding of the interplay between information processing and evidence gathering, and whether strategies in the former have analogs in the latter (or vice versa). For example, confirmation bias has analogs in both evidence seeking (selecting sources you expect to have content that aligns with your beliefs) and information processing (down-weighting the content at odds with your beliefs). While it is possible that how people select relevant sources is a different cognitive process than their information processing of first-order data, the two may reflect similar overall strategies. In the *Discussion* section, we return to this and its importance for designing tailored communication so as to avoid fallacious inferences.

What we would like to do is offer people, in a controlled setting, a wider and more fine-grained selection of evidence that spans both gathering and processing research. For the processing side, we will use “statistical” to mean the entries in a contingency table (but unprocessed, so to speak). This enables us to detect the possibilities of processing heuristics. On the information gathering side we will use “deference” to mean sources where information has been pre-processed or given an interpretation (e.g., a recommendation) and seen as potentially relevant without the presumption that subjects see the sources as experts. Now if we give subjects even just 12 options to select from across these two sides, we face the problem of combinatorial explosion that the typologies of evidence are meant to make tractable, especially if we consider the full pathway of gathering information and its processing (e.g., by making a judgment about causal efficacy). In the next section we illustrate an analytical approach for handling this challenge.

### Supervised clustering-based typology of evidence

At a high level of description, we meet the analytical challenge with a two-pronged approach. The first prong is that we replace the *category-driven* methodology of selecting evidence categories in advance with a *cluster-driven* approach. The second prong is to generate the clusters in a meaningful and non-arbitrary way by using some criterion to generate a “label” and a means of weighting how much different information *sources* (not the information itself) predict said label. The resulting clusters can then be compared *post-hoc* to prior evidence categories in existing typologies.

More concretely, consider a context where people are asked to assess a causal claim based on available fine-grained pieces of evidence. Here a *supervised* clustering approach [26–28] can be used to identify empirical patterns in evidence gathering behaviors. Supervised clustering allows us to combine evidence accessed (requested) by people with their evaluation of the causal claim they were asked to assess (the “label”). This clustering is based on the SHapley Additive exPlanation (SHAP) values of each piece of evidence [29,30]. Specifically, the SHAP value is the amount that a particular feature (an evidence in our case) marginally contributes to the prediction of an individual’s assessment beyond a baseline average prediction [31]. Feeding these SHAP values to a traditional clustering algorithm then gives *empirically-driven* groups in which respondents have a strong tendency to have similar assessments.

In other words, we will show below how supervised clustering can be used to mitigate the need of evidence typology priors. The SHAP methodology has the advantage of both reducing the role of the researcher’s priors (reduction of fine-grained choices to broad categories, or use of predefined broad types of evidence) and linking evidence gathering to information processing. We shall further argue that this supervised clustering approach approximately integrates the whole pathway from evidence seeking to information processing and decision-making (see section *Discussion*).

### The role of cognitive heuristics in evidence gathering

To illustrate the fruitfulness of the SHAP methodology for bridging work on evidence gathering and information processing, we select a particular application. Here, we want to get a better understanding of how people navigate situations where they can select from both various “expert” sources and first-order data (e.g., values of cells in a 2×2 contingency table). More specifically, we are interested in whether two common heuristics in drawing (fallacious) causal inferences from 2×2 contingency tables [24,23] have analogs in evidence gathering, even when this information processing can be deferred to outside sources. The ‘base rate neglect heuristic’ only considers the treatment group, comparing the number of positive outcomes to the number of negative outcomes. The ‘confounders neglect heuristic’ compares the number of positive outcomes between both the treatment group and the control group, but ignores the number of negative outcomes across both. Both heuristics are only considering *partial* statistical data from a contingency table, whereas *full* consideration would be a comparison between the ratio of positive to negative outcomes in the treatment group to the ratio in the control group.

Our goal is to exploit the SHAP methodology to identify patterns in evidence gathering behavior (e.g., base rate neglect, confounders neglect), and assess their association with psychological variables that underpin information processing. We target the link between patterns of evidence gathering and *cognitive reflection*, i.e., the conscious and deliberate reconsideration of an initial intuition [32]. In particular, considering a body of work on the determinants of standards of evidence [33–41], we conducted a statistical test on the following hypothesis: *H*_1_: a group of individuals with higher level of cognitive reflection is more likely to rely on full statistical data than a group of individuals with lower cognitive reflection. Indeed, individuals with *higher level of cognitive reflection* are expected to have a higher probability to examine claims using deliberative and reflective reasoning, rather than exclusively relying on heuristics for information processing. Evidence of such association would suggest that predisposition to use cognitive heuristics affects evidence seeking behavior.

### An observational setup for information gathering and processing

Given our study of interest and the analytical approach we wish to illustrate, we need an observational setup that balances several design choices. We want a domain where people have skin in the game (e.g., health) – in contrast to more abstract topics wherein identity and group signaling can be overly weighted, like evolution, or more distant long-ranging issues like climate change. Relatedly, we want to minimize the role of priors (evidential or motivated) and the potential for confirmation bias, and heighten the signal of new information as it goes through the pathway of selection, processing, and decision making. We also want to allow for the possibility that respondents consider sources of secondary relevance – you might ask an expert first, but if they don’t know or only have partial answers, you may be willing to find supplementary information from other sources. And to the extent that such information is statistical data, it needs to be sufficiently fine-grained in order to accommodate the possibility of cognitive heuristics.

The key strategy behind our specific design was to mask the content of evidence in the form of queries. Subjects select which of these are relevant, and only then are provided the content. More specifically, in our observational setting subjects are asked to assess the effectiveness of a new nasal spray against COVID-19. To help them in their assessment, we offer them 12 possible pieces of evidence of multifarious typology. Some examples are: (*ev*_1_) “How many of the company’s employees have used the nasal spray and got infected with COVID-19?” and (*ev*_6_)“What does the CDC say about the effectiveness of the nasal spray?” If selected, the answers, respectively, are “126 company employees have used the nasal spray and got infected with COVID-19” and “According to the CDC, there is not enough research to say whether the spray is effective against COVID-19 or not” (see Table 1 for the full list). Twice subsequently, respondents are given the opportunity to select more evidence after viewing the details of what they already selected. We then close the loop from their evidence gathering to information processing by soliciting their assessment about the spray’s effectiveness.

**Table 1:** Evidence available to respondents (question, answer, and abbreviation on figures).

**Notation**	**Evidence proposed to respondents**	
*ev*_1_	*Question:*	How many of the company’s employees have used the nasal spray and got infected with COVID-19?
	*Answer:*	126 company employees have used the nasal spray and got infected with COVID-19.
	*Abbreviation on figures:*	“employees used infected”
*ev*_2_	*Question:*	How many of the company’s employees have used the nasal spray and did NOT get infected with COVID-19?
	*Answer:*	84 company employees have used the nasal spray and did NOT get infected with COVID-19.
	*Abbreviation on figures:*	“employees used not infected”
*ev*_3_	*Question:*	How many of the company’s employees have NOT used the nasal spray and got infected with COVID-19?
	*Answer:*	352 company employees have NOT used the nasal spray and got infected with COVID-19.
	*Abbreviation on figures:*	“employees not used infected”
*ev*_4_	*Question:*	How many of the company’s employees have NOT used the nasal spray and did NOT get infected with COVID-19?
	*Answer:*	88 company employees have NOT used the nasal spray and did NOT get infected with COVID-19.
	*Abbreviation on figures:*	“employees not used not infected”
*ev*_5_	*Question:*	What does the nasal spray do in the human body according to the manufacturer?
	*Answer:*	According to the manufacturer, the nasal spray contains antimicrobial ingredients which prevent the virus from replicating in the human body.
	*Abbreviation on figures:*	“manufacturer info”
*ev*_6_	*Question:*	What does the CDC say about the effectiveness of the nasal spray?
	*Answer:*	According to the CDC, there is not enough research to say whether the spray is effective against COVID-19 or not.
	*Abbreviation on figures:*	“cdc info”
*ev*_7_	*Question:*	Has the nasal spray been approved by the Food and Drug Administration for use against COVID-19?
	*Answer:*	According to the Food and Drug Administration (FDA), the nasal spray is “Generally Recognized as Safe” for use in the human body.
	*Abbreviation on figures:*	“fda approval”
*ev*_8_	*Question:*	What company manufactures the nasal spray?
	*Answer:*	The nasal spray manufacturer is called “Smith & Coudrine” — a relatively new Canadian pharmaceutical company.
	*Abbreviation on figures:*	“company name”
*ev*_9_	*Question:*	What do medical doctors in the U.S. say about the effectiveness of the nasal spray?
	*Answer:*	Most medical doctors in the U.S. say that existing research on the effectiveness of the nasal spray is inconclusive.
	*Abbreviation on figures:*	“us doctors opinion”
*ev*_10_	*Question:*	What do clinical trials show about the effectiveness of the nasal spray?
	*Answer:*	There are a number of ongoing clinical trials which attempt to assess the effectiveness of the nasal spray. These trials have not been completed yet.
	*Abbreviation on figures:*	“clinical trials”
*ev*_11_	*Question:*	What do animal studies show about the effectiveness of the nasal spray?
	*Answer:*	Animal studies of mice have shown that the nasal spray can activate specific cells that are part of their immune system.
	*Abbreviation on figures:*	“animal studies”
*ev*_12_	*Question:*	What do public health authorities in India say about the effectiveness of the nasal spray?
	*Answer:*	Public health authorities in India have stated that the nasal spray reduces people’s likelihood of getting infected with COVID-19.
	*Abbreviation on figures:*	“india health authorities”

Masking the evidentiary content behind its relevant query has several advantages. First, we can observe what people take to be relevant *sources* as separate from the content; in regards to testimony, this reflects whose opinion is relevant. While we cannot ultimately say how some content was used or weighted in someone’s information processing, we can at least say they ignored that which they never selected in the first place. But also, we can establish counterfactuals about what subjects would have deemed as relevant content or relevant sources (in cases where the content itself was “empty” so to speak, as in *ev*_6_ above).

Second, we can control content to detect known heuristics in information processing (as successfully deployed in the study of motivated numeracy [24]) to track them across evidence gathering behaviors. For example, the content of *ev*_1_ through *ev*_4_ is equivalent to a 2×2 contingency table, where the implied base rate of infection is 80%, and that of the test group is 60%; the combined contents of $ev_{1}-ev_{4}$ point in the direction that the nasal spray is effective. Two common heuristics, however, would point fallaciously in the direction that the nasal spray is not effective: the base rate neglect heuristic would only consider *ev*_1_ and *ev*_2_ to be relevant (i.e., 60%), and the confounder neglect heuristic would only consider the positive effect outcomes *ev*_2_ and *ev*_4_ as relevant (84:88, i.e., similar numbers of not getting infected).

Third, we can nudge people towards information processing by having incomplete or irrelevant content from certain sources that people may attempt to defer information processing to (as illustrated above by *ev*_6_). Deference is epistemically efficient, especially given how highly interdependent we are – why spend the effort to process information if it may have already been processed? (Of course, this can come with bad consequences [25]). But in the absence of such convenience, we may be willing and able to do the information processing ourselves (e.g., interpreting a 2×2 contingency table).

From using this observational setting and applying the SHAP methodology, we find that the use of the cognitive heuristic related to base rate neglect drives statistical evidence gathering behavior *under similar patterns of deference behavior*. We do not, however, find such an analog for the confounder neglect heuristic. We thereby track a key thread in the natural emergence of diverse evidence seeking and processing strategies and underscore the importance of cognitive abilities in shaping approaches to evidence gathering. In light of our finding, we suggest an alternative delineation of evidence types, which we describe in the discussion.

## Methods

### Study design

An online survey was conducted in the United States (U.S.) from August 19, 2024 to August 20, 2024. First, we designed a questionnaire containing a wide range of demographic questions, items about a respondent’s socio-political characteristics, and a survey module that captures people’s evidence gathering behaviors (described in more detail below). Prior to running the survey, we obtained exemption for this research under category 2 at 45 CFR 46.101(b)(2) from the Institutional Review Board of the University of Idaho [Project Number: 24–136]. Second, we programmed our survey on the online platform Qualtrics [42]. A total of 2,951 participants (minimum age: 18 years old) were recruited using the online platform Prolific Academic Ltd [43]. This platform uses a *voluntary response sampling* which is a non-probability sampling method where participants self-select to join the survey. For this study, participants recruitment occurred through open invitations by organic word of mouth, social media sharing, and outreach initiatives [43]. The sample was designed to approximate the current population distribution in the U.S. on the dimensions of age, gender, and political affiliation. This goal was largely achieved (see Table I in S1 Appendix). Our sample matches the U.S. population breakdown quite well in terms of Age, Gender, and Republican partisan affiliation. By contrast, Democrats are slightly over-represented in our sample (33% vs. 28%) whereas Independent are slightly under-represented (38% vs. 43%).

All survey respondents first read a short study introduction that informed them about the purpose of our survey and their rights as research participants. Informed consent was then obtained digitally. In particular, individuals read that “by completing and submitting your responses you certify that you are at least 18 years of age and agree to participate in the above described research study.”

The demographic characteristics of participants in this study are summarized in Table 2. Respondents had median age class of [45, 54] years, consisted of 52.3% women, and 73.3% self-identified as White. The median educational attainment was “four-year college or university degree”.

**Table 2:** Demographic characteristics of respondents.

Demographics	Mean (SD) [range]	n	Demographics	Mean (SD) [range]	n
Age	3.59 (1.60) [1, 6]	–	Income level	3.43 (1.69) [1, 7]	–
1: 18–24		350	1: < $25,000		365
2: 25–34		535	2: $25,000 to $49,999		700
3: 35–44		525	3: $50,000 to $74,999		555
4: 45–54		466	4: $75,000 to $99,999		453
5: 55–65		677	5: $100,000 to $149,999		505
6: 65 or older		385	6: $150,000 to $199,999		216
Gender	0.48 (0.50) [0, 1]	–	7: $200,000 or more		144
1: Man		1399	Race	0.74 (0.44) [0, 1]	–
0: Woman		1539	1: White		2164
Education level	5.32 (1.63) [1, 8]	–	0: Non-white		774
1: Less than HS		15	CRT-7 score	3.15 (2.31) [0, 7]	–
2: Incomplete HS		26	Ideology	2.88 (1.15) [1, 5]	–
3: HS Graduate		373	1: Very liberal		346
4: Some College, ND		688	2: Liberal		755
5: Two Year AS		331	3: Moderate		838
6: Four Year CUD		956	4: Conservative		622
7: Some PS		84	5: Very Conservative		238
8: Postgraduate PD		465			

SD = standard deviation; *n* = number of respondents; HS = high school; ND = no degree; AS = associate’s degree;

CUD = college or university degree; PS = postgraduate school; PD = professional degree.

### Data collection

We presented each participant with information about a realistic but ultimately fictitious new medical treatment. Specifically, participants were instructed to gather information from a designated “evidence bank” to assess the effectiveness of a new antiviral nasal spray marketed as a potential protection against contracting COVID-19. At the beginning of the survey, respondents first read some contextual information. In particular, we told survey takers that the “nasal spray does not require a prescription and is available over-the-counter in the United States. The manufacturer says that using the spray 2-3 times over the course of a day substantially reduces the likelihood of getting sick with COVID-19.” Next, we gave people information on a small-scale public health initiative. Respondents were informed that “a few months ago, a major midwestern company offered this new nasal spray for free to all of its employees. Last week, the firm conducted a survey of its employees. In this poll, people were asked if they used the nasal spray as directed and if they have since gotten sick with COVID-19.”

After this introduction, survey-takers were told that they should now assess whether taking the nasal spray influences people’s likelihood of getting infected with COVID-19. We explained that they would be presented with various pieces of evidence (*ev*) to assist in making this judgment. Participants could review as many *ev* as they wished. After they felt like they had enough evidence, they would be able to provide their final assessment.

The “evidence bank” in our study contained 12 types of information (see Table 1). We designed this bank to include both fine-grained evidence of “statistical” type and evidence of “deference” type. The content of *ev*_1_ through *ev*_4_ is equivalent to a 2×2 contingency table (“statistical” data). Evidence *ev*_5_ through *ev*_12_ propose information from specified deference sources such as the manufacturer of the nasal spray, the Centers for Disease Control and Prevention (CDC), the Food and Drug Administration (FDA), medical doctors in the U.S., scientists (clinical trials and animal studies), and “public health authorities in India”. We point to some limitations of this evidence bank in the section *Discussion*.

Respondents could select which *ev* they wanted to retrieve with each option clearly describing the nature of the *ev* without indicating whether it supported or opposed the effectiveness of the nasal spray. After individuals clicked on the desired *ev* (minimum 0, maximum 12), the survey would display the corresponding information to them. We term the result of this first round of selection *sources of primary relevance*, as they represent what respondents identify as the most relevant evidence for making an informed assessment. Subsequently, respondents could decide if they had enough information to make a final evaluation or if they wanted to consult the *ev* bank again. If they chose to return to the *ev* bank, they choose to display the same 12 types of information. Then, subjects had the option of providing their final assessment or returning to the *ev* bank one final time. We term the result of these additional rounds of selection *sources of secondary relevance*.

Following the presentation of *ev*, individuals provided their assessment of the effectiveness of the nasal spray. First, they responded to a prompt that asked them which of the following statements was better supported by the *ev* that they had reviewed. Answer options were (1) “Taking the nasal spray reduces people’s likelihood of getting infected with COVID-19” (31.4%); (2) “Taking the nasal spray does NOT reduce people’s likelihood of getting infected with COVID-19” (55.2%); (3) “I don’t know” (13.5%). Second, people were asked to indicate how certain they were of their assessment. Here, answer options ranged from (1) “Not certain at all” to (4) “Very certain”.

In order to test the hypothesis *H*_1_, we rely on the widely used Cognitive Reflection Test (CRT-7) [20,35,40] to capture people’s levels of cognitive reflection. Specifically, respondents are presented with seven questions, each designed to elicit an intuitive (yet incorrect) answer. Respondents have to engage in deliberate, reflective thinking to arrive at a correct answer. For example, “Jerry received both the 15th highest and the 15th lowest mark in the class. How many students are in the class? (a) 28, (b) 29, (c) 30, (d) 31”. The intuitive answer is (c) obtained from 15 + 15 = 30. However, since Jerry himself is counted twice in that addition, we need to subtract 1, so the correct answer is (b). All questions that are part of the CRT-7 battery are provided in S1 Appendix (section *Supplement to methods*). The total number of correct answers per respondent is termed *CRT-7 total score*. The average CRT-7 total score in our sample is 3.15 (see Table 2).

Political ideology shapes how people engage with evidence [39,41]: conservatives tend to seek new evidence less often than liberals [37]. Because our observational setup was designed to minimize the role of priors and confirmation bias, we do not expect political ideology to confound cognitive reflection. We nonetheless asked respondents to place themselves on a 5-point ideological spectrum (see Table 2) and included this as a potential confounding variable in the statistical test of *H*_1_. In our sample, 12.4% of respondents self-identified as “very liberal”, 27.0% as “liberal”, 30.0% as “moderate”, 22.2% as “conservative”, and 8.5% as “very conservative”.

Among the 2,951 recruited participants, 42 did not select any *ev* and all selected the response option “I do not know”. These respondents were considered uninterested in the assessment of the effect of the nasal spray based on the provided *ev*. They were thus all excluded from all statistical analyses which considered the remaining *N* = 2909 participants.

Most *ev* (9/12) were selected by more than half of respondents as *source of primary relevance*. Only 25% (i.e., 683/2730) of respondents who could have selected more *ev* have taken the opportunity to gather more evidence. Indeed, each *ev* was a *source of secondary relevance* for less than 8% of respondents (see details in S1 Appendix, section *Supplement to results*). As a result, we used the total *ev* selected by respondents in our analyses, aggregating *sources of primary relevance* and *sources of secondary relevance*.

### Statistical analysis

We considered an analysis pipeline with three steps. The *first* step described how the *ev* selected by respondents explain their assessments of the effectiveness of the nasal spray using SHapley Additive exPlanation (SHAP) values [30]. The *second* step identified empirical groups of respondents (with similar evidence selections for the purpose of assessing the effectiveness of the nasal spray) and the between-group structure. The *third* step consisted in testing the hypothesis *H*_1_, that is, determining whether CRT-7 total score predicts identified empirical group memberships.

#### SHAP value calculation.

To describe how the *ev* selected by respondents explain their assessments, we followed established research practices [44,45] and combined the evaluation of the effect of the spray and the self-reported uncertainty by each respondent to define an integer response *Z*. Explicitly, the variable *Z* is negative when a respondent answered “spray does NOT reduce infection rate”, positive when a respondent answered “spray reduces infection rate”, and zero when a respondent answered “I don’t know”. The absolute value of *Z* is derived from the level of self-reported uncertainty: |*Z*| = 1 if respondent is “not certain at all”, |*Z*| = 2 for “somewhat certain”, |*Z*| = 3 for “fairly certain”, and |*Z*| = 4 if respondent is “very certain”. The resulting variable *Z* ranges from Z=−4 (“very certain” that the “spray does NOT reduce infection rate”) to *Z*=+4 (“very certain” that the “spray reduces infection rate”), with the middle point *Z* = 0 indicating no confidence. The set of predictors for *Z* is the 12-column binary matrix representing all *ev* selected by respondents.

Next, we built a statistical model to predict *Z* given the *ev* selected by a respondent. As candidate predictive model, we considered the multiple linear regression model and four competitive alternatives including Generalized Additive Model (GAM) [46], ordinal logistic regression [47], XGBoost tree regressor and XGBoost ordinal classifier [48]. As performance metrics, we computed the Mean Absolute Error (*MAE*), the Root Mean Square Error (*RMSE*) and the Spearman rank correlation ρ between observed value and predicted value using a 30% testing subset (*n* = 873) of the whole data (*N* = 2909) after training the model on a 70% training set (*n* = 2036). Table 3 compares candidate models fitted to predict respondents’ assessment of the effectiveness of the nasal spray. Based on all considered performance measures (*MAE*, *RMSE*, and Spearman ρ), the XGBoost tree machine provided the best predictive model for the effectiveness assessment data.

**Table 3:** Performance of multiple linear regression (MLR), generalized additive model (GAM), ordinal logistic regression (OLR), XGBoost tree regression (XTR), and XGBoost ordinal classifier (XOC) for predicting respondents’ assessment of the nasal spray’s effect after the final round of evidence selection.

Model	Performance measures		
	*MAE*	*RMSE*	Spearman ρ
MLR	1.97	2.26	0.27
GAM	1.97	2.26	0.27
OLR	1.97	2.27	0.27
XTR (d^*^ =1)	1.97	2.26	0.27
XTR (*d* = 2)	1.96	2.24	0.29
XTR (*d* = 3)	1.97	2.35	0.26
XOC (*d* = 1)	1.97	2.27	0.27
XOC (*d* = 2)	1.97	2.30	0.27
XOC (*d* = 3)	1.97	2.33	0.23

The predictors in each model are the 12 pieces of evidence selected or not (binary) by respondents. ^*^ *d* = “max_depth”, hyperparameter (in tree models) that controls the maximum number of levels or splits the tree can have (a high value can learn more complex patterns while a low value can prevent overfitting). *MAE* = Mean Absolute Error; *RMSE* = Root Mean Square Error; Spearman ρ is the Spearman rank correlation between observed value and predicted value.

Then, we used the SHAP methodology to deconstruct each individual prediction of the XGBoost machine into a sum of contributions from each of the predictors [30]. Specifically, the SHAP value is the amount that a particular feature (an evidence in our case) marginally contributes to the prediction of an individual’s response (assessment *Z* in our case) beyond a baseline average prediction [31]. Mathematical description and computation of SHAP values in Python version 3.10.13 [49] are detailed in S1 Appendix (section *Supplement to methods*). The mean absolute SHAP (MAS) value was computed to identify the most important *ev*.

#### Clustering on SHAP values.

The *second* step identified empirical groups of respondents (with similar evidence selections for the purpose of assessing the effectiveness of the nasal spray) and the between-group structure. To that end, we used agglomerative Hierarchical Clustering (HC) based on Euclidean distance and Ward’s minimum-variance algorithm [50] in R version 4.5.1 [51] to produce a nested hierarchy among potential groups in the data. Specifically, supervised clustering [26,28] (in which respondents in a cluster have a strong tendency to have similar *Z* values) was used because it provided more compact and stable clusters as compared to traditional unsupervised clustering (see details in S1 Appendix, section *Supplement to methods*). We performed a Principal Components Analysis (PCA) in R to visualize the hierarchy of potential clusters using the main information.

#### Logistic regression.

The *third* step consisted in testing the hypothesis *H*_1_, that is, determining whether CRT-7 total score predicts identified empirical group memberships. Specifically, we fitted a Multinomial Logistic Regression (MNLR) model in R to the group membership against CRT-7 total score, with demographic characteristics including gender (man or woman), age, race (white or not), education level and political ideology as potential confounding variables. See details including treatment of missing values and model selection in S1 Appendix (section *Supplement to methods*). An alternative approach to test *H*_1_ is to fit a binary logistic regression to the selection or not of the full first-order evidence ($ev_{1}-ev_{4}$) against the same predictors. The result from this binary logistic regression is consistent with the MNLR result (see S1 Appendix, section *Supplement to methods*). We focus on the MNLR result since it integrates other information gathered by respondents ($ev_{5}-ev_{12}$). All data and codes used for analyses are publicly available via OSF at https://osf.io/78kxp/?view_only=fd5aafd8a4ab4e1ca6cf679b3164bccb.

## Results

The bank of the 12 queries that respondents could select from and reveal the respective answers to (indexed *ev*_1_ to *ev*_12_) is available in Table II in S1 Appendix. All subjects could see all the questions of the *ev* query-answer pairs, but only when a subject selects $ev_{j}$ are they then given the content of the answer part of $ev_{j}$. The *ev* selected by respondents and their assessments *Z* are also summarized in Fig II and Fig III in S1 Appendix.

### Empirical patterns in evidence gathering behaviors

Our results indicate that meaningful groups of individuals with differentiated evidence gathering behaviors can be empirically delineated without resorting to standard pre-defined frameworks. We employed a supervised clustering method which consisted in first determining the contributions of *ev* selected by respondents to their assessments *Z* using SHAP values, and then applying a HC algorithm to them.

#### SHAP values.

**Fig 1:**
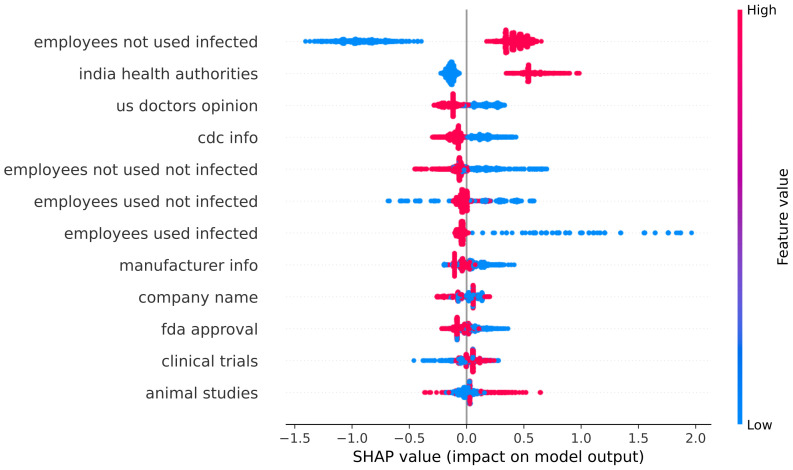
Distribution of SHapley Additive exPlanation (SHAP) values. Each row in the summary plot represents an $ev_{j}$ (j=1,⋯,12) and each dot in a row represents a respondent (*N* = 2909). The $ev_{j}$ are abbreviated for conciseness (see full list in Table II in S1 Appendix). The color of the dot indicates whether a respondent selected $ev_{j}$ and was exposed to the answer (red) or did not select it (blue). The position of a dot (respondent) on the x-axis indicates its SHAP value, which measures the marginal contribution of a piece of evidence to the model prediction for the corresponding respondent. For instance, if respondents who selected $ev_{j}$ are associated with a large positive SHAP value, then this would suggest that the selection of $ev_{j}$ has a large positive contribution to the model prediction (an example of such an *ev* is *ev*_3_ = “employees not used infected”). Conversely, if respondents who selected $ev_{j}$ are associated with a negative SHAP value, this would suggest that the selection of $ev_{j}$ has a negative contribution to the model prediction (an example is *ev*_9_ = “us doctors opinion”). The height of the plots in each row indicates how many respondents are associated with that specific SHAP value.

Fig 1 displays the distributions of SHAP values which represent additive contributions of the choice of different *ev* to the assessment *Z* by a respondent. The selection or not of *ev*_3_ = “352 company employees have NOT used the nasal spray and got infected with COVID-19” and *ev*_12_ = “Public health authorities in India have stated that the nasal spray reduces people’s likelihood of getting infected with COVID-19” were the most discriminating choices, with Mean Absolute SHAP (MAS) values: MAS=0.59 for *ev*_3_ and MAS=0.24 for *ev*_12_ (see MAS details in Fig IV in S1 Appendix).

We observe that a high SHAP value for each of these two *ev* is consistently associated with the selection of the *ev*. In other words, respondents who selected these two *ev* were the most likely to confidently assess the spray as effective, and conversely, those who did not select these two *ev* were the most likely to confidently assess the spray as not effective. In accordance with that, the results of a Correspondence Analysis (CA) on *ev*_3_, *ev*_12_, and *Z* (see CA map in Fig V in S1 Appendix) indicate that respondents who selected none of *ev*_3_ and *ev*_12_ mostly evaluated the spray as not effective and were generally fairly certain (Z=−3) or very certain (Z=−4). In contrast, respondents who selected both *ev*_3_ and *ev*_12_ mostly evaluated the spray as effective and were generally not certain at all (*Z* = 1), and rarely very certain (*Z* = 4).

#### Links between selected pieces of evidence.

It is worth noticing that the selection of *ev*_3_ is highly correlated with the selection of *ev*_2_ = “84 company employees have used the nasal spray and did NOT get infected with COVID-19” and *ev*_4_ = “88 company employees have NOT used the nasal spray and did NOT get infected with COVID-19” (see Multiple Correspondence Analysis (MCA) results in Fig VI in S1 Appendix). Indeed, a respondent who has selected *ev*_3_ has a 93% conditional probability to have also selected *ev*_2_ and 86% for *ev*_4_. Compounded with a 99% conditional probability to have also selected *ev*_1_ = “126 company employees have used the nasal spray and got infected with COVID-19”, such a respondent likely has full first-order evidence to (correctly) assess the effectiveness of the nasal spray. Likewise, a respondent who has selected *ev*_12_ has high conditional probability to have also selected *ev*_1_ (100%), *ev*_2_ (100%), *ev*_3_ (87%) and *ev*_4_ (84%). Such a respondent likely not only has full first-order evidence, but also has conclusive evidence by deference to assess the nasal spray as effective against COVID-19.

#### Tree of hierarchy.

**Fig 2:**
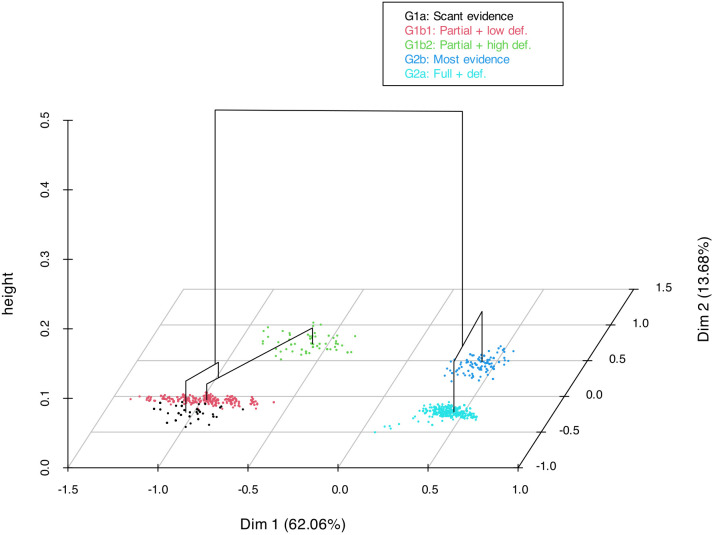
Results of supervised hierarchical clustering (HC). The plot shows the tree of hierarchy and five clusters of respondents in the first two Principal Components (PC). Each dot on the graphic represents a respondent (*N* = 2909) and colors indicate group memberships. The indicated labels of clusters were derived from the characterization of the five clusters based on the tree of hierarchy and the proportions of respondents who selected each piece of evidence in each cluster. Note that only four clusters are clearly separated because the third PC is not shown (see Fig VII in S1 Appendix for a visualization in the first and third dimension). The tree of hierarchy however clearly shows that the group “G1b2: Partial + high def.” (green) is closer to “G1b1: Partial + low def.” (red) than “G1a: Scant evidence” (black) is to G1b1.

Fig 2 displays the tree of hierarchy among groups of respondents resulting from HC in the principal plane accounting for 76% of the variability in SHAP values. Four well-separated groups of respondents can be visually spotted in the principal plane. The tree of hierarchy however indicates five distinct groups (this becomes obvious when the third principal dimension is also considered, accounting for 84% of variability, see Fig VII in S1 Appendix).

#### Group behaviors.

**Fig 3:**
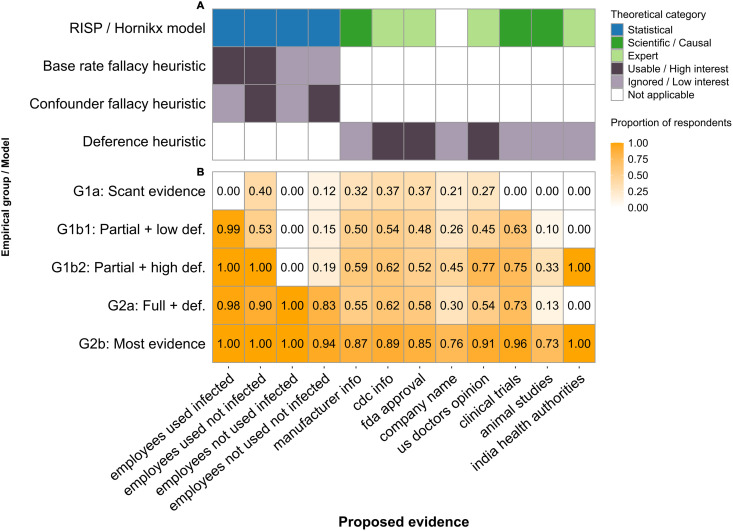
Heatmap comparing theoretical and empirical categorizations of evidence. **A** shows the theoretical categorizations of the pieces of evidence proposed to respondents. **B** shows the evidence selection patterns among five empirically defined groups of respondents using supervised clustering. The columns represent the 12 pieces of evidence (*ev*) proposed to respondents (*N* = 2909). Each row in **A** represents a theoretical model: a superposition of Hornikx model and the Risk Information Seeking and Processing (RISP) model, two heuristics using base rate fallacy (ignoring control first-order data) and confounder fallacy (ignoring people who got infected, irrespective of the use or not of the nasal spray), and a deference heuristic valuing only official stamp or local authorities. Each row in **B** represents a group of respondents on Fig 2 (“def.” in group labels means “deference”) and the number in each cell is the proportion of respondents who selected the corresponding *ev*. The group labels refer to the characterization of the five clusters based on the tree of hierarchy and the proportion of respondents who selected different *ev* in each cluster. “G1a: Scant evidence” respondents (first row) mainly use only one first-order datum, have a low evidentiary standard, or use a heuristic as they were not interested in positive COVID-19 test results nor in the outside sources indirectly appealing to first-order data (*ev*_10_ = “clinical trials” and *ev*_11_ = “animal studies”). “G1b1: Partial + low def.” respondents have a partially associative/statistical standard (not interested in the control group, i.e., employees who did not use the nasal spray) and show moderate interest in what other sources have to say. “G1b2: Partial + high def.” is similar to “G1b1” (not interested in the control group data) but respondents see increased relevance of other sources. “G2a: Full + def.” respondents put high emphasis on all first-order data, requested some outside sources, but were less interested in sources such as *ev*_8_ = “company name”, *ev*_11_ = “animal studies” or *ev*_12_ = “india health authorities”. “G2b: Most evidence” respondents have the most expansive body of evidence from multiple sources and types of data.

For each of the five empirical groups of respondents shown on Figs 2 and 3, depicts the proportion of respondents who selected each proposed *ev*, along with evidence categorizations based on four theoretical models. These include a superposition of Hornikx model [17] and the Risk Information Seeking and Processing (RISP) model [16], two heuristics using base rate neglect and confounder neglect [52], and a deference heuristic valuing only official stamp or local authorities [53,54].

As expected from a supervised clustering, differences between clusters are mainly related to *ev* that best explained respondents’ assessments of the effectiveness of the nasal spray. First, *ev*_3_ separated respondents into two super-clusters G1 (respondents who did not select *ev*_3_) and G2 (respondents who selected *ev*_3_). By comparison with extant models of evidence types, these two groups roughly correspond to respondents that make use of partial statistical data (G1, *n* = 919 respondents, 32%) and respondents with fully statistical standard of evidence (G2, *n* = 1990 respondents, 68%).

Within group G1, a first split distinguishes respondents who did not select *ev*_1_ (see above), *ev*_10_ (“There are a number of ongoing clinical trials which attempt to assess the effectiveness of the nasal spray. These trials have not been completed yet.”), and *ev*_11_ (“Animal studies of mice have shown that the nasal spray can activate specific cells that are part of their immune system.”) (G1a) from those who selected any of these three *ev* (G1b). G1a respondents were mostly only interested in one statistical source (*ev*_2_) and not interested in the outside sources which directly appeal to first-order data (*ev*_10_ and *ev*_11_). On the one hand, G1a respondents were not interested in *ev*_12_. On the other hand, G1b is a mixture: G1b1 who did not select *ev*_12_ and G1b2 who selected *ev*_12_. Within the group G2, *ev*_12_ is the main discriminant of respondents with selective deference (G2a, *n* = 1390 respondents, 48%) and respondents who selected almost all *ev*.

Overall, the groups G1a (*n* = 92 respondents, 3%) and G1b2 (*n* = 91 respondents, 3%) are minorities and most of respondents are shared between the groups G2a (*n* = 1390 respondents, 48%), G1b1 (*n* = 736 respondents, 25%) and G2b (*n* = 600 respondents, 21%). In particular, G2a and G1b1 are groups of individuals that are selective in their evidence choices, with similar interests in deference sources, but different interests in first-order data: most G1b1 respondents did not request control cases (*ev*_3_ and *ev*_4_) while most G2a respondents requested full first-order data. In regard to assessments, a G1b1 individual has a 73% probability (95% confidence interval: *CI*_95%_=[70%, 76%]) to assess the nasal spray as not effective. The corresponding probability is 22 percentage points lower (51%; *CI*_95%_=[48%, 54%]) for a G2a individual. For both groups, the probability of being undecided is about 13% (see full summary statistics in Table VIII in S1 Appendix). In accordance with these statistics, G1b1 individuals mostly assessed the nasal spray as not effective with high confidence (Z=−3 and Z=−4, i.e., fairly certain or very certain) while G2a individuals assessed the nasal spray more in the direction of effectiveness (see CA map in Fig VIII in S1 Appendix).

### Cognitive reflection and political ideology

Our results were consistent with *H*_1_. We report here the MNLR modeling the probability for an individual to belong to each of the five empirical groups as a function of the Cognitive Reflection Test (CRT-7) score and a political ideology (conservatism) score.

**Fig 4:**
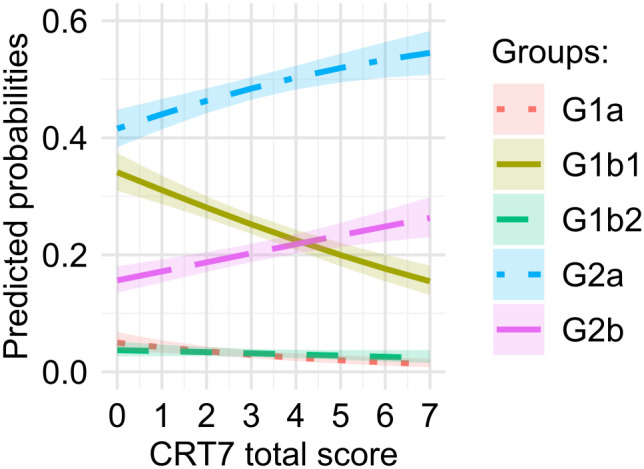
Results of multinomial logistic regression. The curves show predicted probabilities for a respondent to belong to each of five empirical groups of respondents (*N* = 2909) as a function of CRT-7 total score. The shaded region along each curve represents the 95% confidence band. These predicted probabilities do not depend on the reference empirical group used in the regression model (“G1a: Scant evidence”). The indicated groups labels correspond to those described under Fig 3.

After controlling for relevant demographic factors, the CRT-7 total score ($\chi_{4}^{2}=81.04$, *P* < 0.001) of respondents significantly affected group membership whereas the ideology score ($\chi_{4}^{2}=5.94$, *P* = 0.204) was not significant (see Table IX in S1 Appendix). Fig 4 depicts the predicted probabilities to belong to each of the five groups as a function of CRT-7 total score. We observe that the probabilities to belong to the groups G2a and G2b (respondents requesting full first-order statistical information and some deference sources) increase with the CRT-7 total score. In contrast, the probability to belong to the group G1b1 (respondents requesting only one or two first-order data and some outside sources) decreases with the CRT-7 total score. The probability to belong to minority groups G1a or G1b2 is low and slightly decreases with the CRT-7 total score. Overall, increasing CRT7 score is associated with increasing probability to be in groups with a fully statistical standard of evidence and decreasing probability to be in groups using partially statistical standard of evidence (see CA map in Fig IX in S1 Appendix). Altogether, these findings provide support for hypothesis *H*_1_.

## Discussion

In this study, we used original online survey data to explore links between what people do with information and their strategies for how they select information in the first place. We focus specifically on how people navigate data that need processing versus deference to outside sources. Our observational framework used a hypothetical new health product to limit influence from prior beliefs and motivated reasoning by respondents. Our setup did not include explicitly contradictory pieces of evidence, except insofar as some information processing styles are concerned, a signal we could detect in our analysis.

### Related work

Previous research relied on theoretical delineations of styles of evidence gathering or sensitivity to sources. While this means that broad categorizations obscure more granular distinctions that drive individual choices, it remains tractable. Our approach instead leveraged supervised clustering, a machine learning technique, to identify empirical types of evidence-seeking behaviors as they occur more naturally. We argue that this supervised clustering approach is an inroad to understanding the pathway from evidence seeking to information processing and decision-making. This argument is supported by results from unsupervised clustering which was not able to separate respondents into clearly demarcated groups (See Tables IV and V in S1 Appendix). In fact, by transforming the original attributes (*ev* selected by individuals) into SHAP values expressing the importance of different *ev* in a unit-less multidimensional space, the SHAP methodology circumvents the challenging problem of explicitly determining feature weightings [27,31]. This amounts to discarding noisy information not contributing to respondents’ assessments. This is comparable to the use of dimension reduction techniques (e.g., Multiple Correspondence Analysis [55]) to discard statistical noise in data and thereby improve pattern identification [56]. However, unlike such techniques which only rely on correlations between attributes, the SHAP methodology mainly distinguishes noise from information precisely useful for predicting individual assessment. By using the prediction of similar answers (*Z*) as the expectation when delineating groups, the supervised clustering procedure isolates and focuses on information effectively processed by participants to make assessments.

The identified empirical evidence-seeking groups overlap with existing categorizations in psychology [14–16] and science communication literature [17–19], but provide more granular distinctions. For example, people can consider evidence of broad first-order type but differ in what specific data is of interest, some being satisfied with partial data and some demanding more comprehensive data. In addition, among people relying on deference, the exact source can vary, for instance, from authorities or organizations with official stamps, to the scientific community and practitioners. Furthermore, people can consider full or partial information from both first-order and deference sources.

It is obviously not possible to directly measure which information respondents actually processed in such an information-diverse setting, let alone to determine the nature of the information processing. We can nevertheless approach this by checking consistent trends in the information subjects gathered (one cannot use information that one has failed to gather), information processing style (via cognitive reflection), and assessment (decision-making). Our results indicate that respondents’ assessments are consistent with the information gathered and expectations of how it would be processed. Indeed, participants who were selective in both first-order data and deference behavior (G1b1) had the highest proportion that made a negative assessment (73%), in accordance with the partial first-order data. A second group of participants (G2a) were similar in their deference behavior, but selected the full first-order data. A correct interpretation of the full 2×2 contingency data points towards a positive assessment, and accordingly, that group saw a decrease in negative assessments (51%).

These results suggest that these groups are processing the information gathered, and difference in assessment is due to difference in gathered information. This implies that the evidence used was first selected because of the *intended* information processing. In other words, respondents who requested partial first-order data were using a heuristic. Specifically, the group of respondents using limited first-order data appear to be using the heuristic that falls prey to the base rate fallacy, which ignores numbers from the control (in contrast to the heuristic that falls prey to the confounder fallacy by ignoring the numbers of negative outcomes). This suggests that, as is well known for confirmation bias, the base rate neglect heuristic in information processing also has an analog in evidence seeking.

As we also observed, this would imply cognitive differences between groups. In particular, respondents with low CRT-7 scores should have high probability to belong to a group using the base rate fallacy while respondents with high CRT-7 score would have high probability to belong to a group using full first-order data. Our conclusion that cognitive reflection style (heuristic/systematic) precedes information gathering is supported by psychological studies which indicate that the CRT is a distinct and measurable trait independent of the mathematical content of its items [57–59]. Furthermore, in the particular context of literature searching during the academic research process, Ford et al. [60] reported suggestive evidence of interactions between information seeking behavior and cognitive styles of postdoctoral researchers. Our results provide further support of patterns in evidence prioritization behavior and cognitive styles of reasoning.

We also found that membership of the empirically delineated groups is not associated with ideological beliefs, as measured by conservatism. While it has been found that conservatism is generally associated with the use of heuristics [61–63], their use can depend on the context or domain in question [9,24]. Our result is consistent with the work of Howe et al. [64] who found no systematic relationship between base rate neglect and conservatism within each individual. The fact that our observational framework considered a new health product to limit effects of priors may have contributed to the absence of this association in our data.

### Bridging evidence seeking and information processing

Our findings overall highlight the natural emergence of diverse evidence seeking and processing strategies through the SHAP methodology and underscore the importance of cognitive abilities in shaping these approaches. By identifying empirical evidence gathering groups and the underpinning cognitive abilities, targeted and tailored health messages can be designed for diverse audiences with different cognitive profiles. The effectiveness of tailored health communication so as to avoid fallacious inferences has been well documented [65,66]. Work in debiasing training and education appears to be effective [67,68]. In particular, our findings reinforce the importance of helping both laypersons and experts overcome base rate neglect [69–71].

Going forward, we advocate for more research that bridges the divide between evidence seeking and information processing. To do so, we recommend a more flexible typology of evidence that can be adapted to contextualized standards of evidence. In particular, we suggest there is significant value in distinguishing between *categorical* and *associative* types of evidence. A categorical standard of evidence relies on either data points that support a particular causal claim, or data points that oppose it. Categorical standards are not necessarily a poor standard. Their appropriateness depends on context. For example, they are used in many routine day-to-day decision making, ranging from matters of taste (e.g., a bad meal can be sufficient to not return to a restaurant) to choices of information sources (e.g., a single mistake by a doctor can be sufficient to mistrust them). They are also used in technical fields, e.g., a mathematical generalization can be falsified by providing a single counterexample. However, categorical standards can be misapplied, as in the case of using only positive outcomes of a treatment to justify claims about trends. (Note that about half of the G1b1 group appear to use a categorical standard when it comes to first-order “statistical” data, while the other half use associative standard). By contrast, associative standards make comparisons between aggregates of multiple types of data points. As hinted at above, associative standards can be either full or partial – depending on whether people collect all evidence necessary to calculate full conditional probabilities in the context of a proposed cause-and-effect relationship or if they rely on heuristic strategies and just compare two quantities of interest.

Our work has a number of limitations to be addressed by future research. We used a voluntary response sampling which is a non-probability sampling method subject to self-selection bias. We limited the over-representation of extreme views by matching the U.S. population in terms of age, gender, and political affiliation. Although respondents were allowed to select all possible pieces of evidence, some evidence types such as anecdotal (including experiential) evidence were not part of our design. To further increase realism, future studies may not only include anecdotal evidence, but also allow respondents to pursue a wider range of evidence, using for instance a web search [72] or AI tool [5]. The complexity of such a design will be further increased by the association between misinformation (e.g., from online sources) and different styles of information processing [73]. As indicated above, our design did not allow direct measurement of what information respondents considered. In regard to that, process tracing methods could be used to triangulate on the unreflective base rate neglect we seem to have detected, for instance, online think aloud protocols [74], eye-tracking [75], or even written reflection prompting [76]. Finally, our results are limited to the health domain, and as intended, to new products or services free of politicized debate and passion. Future work may consider extension to more common health products, or target other domains beyond health.

## Supporting information

S1 Appendix Additional details on methods and results. The appendix provides further information on research design, statistical analysis and results. This includes 9 Tables and 9 Figs.
(PDF)

S1 Fig Fig 1 in scalable vector graphics format. Provides Fig1.svg for scaling without any loss of quality.
(SVG)

S2 Fig Fig 2 in scalable vector graphics format. Provides Fig2.svg for scaling without any loss of quality.
(SVG)

S3 Fig Fig 3 in scalable vector graphics format. Provides Fig3.svg for scaling without any loss of quality.
(SVG)

S4 Fig Fig 4 in scalable vector graphics format. Provides Fig4.svg for scaling without any loss of quality.
(SVG)

## References

[1] Li N, Zeng W, Yin S, Zhao L. How risk communication affects public trust in government: the moderating role of policy expectations. Front Public Health. 2025;13:1557786. doi: 10.3389/fpubh.2025.1557786 40438064 PMC12116495

[2] Correia T. Trust Building in Public Health Approaches: The Importance of a “People-Centered” Concept in Crisis Response. Risk Manag Healthc Policy. 2024;17:1903–8. doi: 10.2147/RMHP.S471250 39104746 PMC11299718

[3] Schlaufer C, Stucki I, Sager F. The Political Use of Evidence and Its Contribution to Democratic Discourse. Public Administration Review. 2018;78(4):645–9. doi: 10.1111/puar.12923

[4] Gille F, Smith S, Mays N. Evidence-based guiding principles to build public trust in personal data use in health systems. Digit Health. 2022;8:20552076221111947. doi: 10.1177/20552076221111947 35874863 PMC9297454

[5] Leung E, Urminsky O. The narrow search effect and how broadening search promotes belief updating. Proc Natl Acad Sci U S A. 2025;122(13):e2408175122. doi: 10.1073/pnas.2408175122 40127267 PMC12002208

[6] Spektor MS, Wulff DU. Predecisional information search adaptively reduces three types of uncertainty. Proc Natl Acad Sci U S A. 2024;121(47):e2311714121. doi: 10.1073/pnas.2311714121 39546563 PMC11588055

[7] Jonas E, Schulz-Hardt S, Frey D, Thelen N. Confirmation bias in sequential information search after preliminary decisions: an expansion of dissonance theoretical research on selective exposure to information. J Pers Soc Psychol. 2001;80(4):557–71. doi: 10.1037//0022-3514.80.4.557 11316221

[8] Druckman JN, McGrath MC. The evidence for motivated reasoning in climate change preference formation. Nature Clim Change. 2019;9(2):111–9. doi: 10.1038/s41558-018-0360-1

[9] Kahan DM. Ideology, motivated reasoning, and cognitive reflection. Judgment and Decision Making. 2013;8(4):407–24.

[10] Bolsen T, Druckman JN, Cook FL. The Influence of Partisan Motivated Reasoning on Public Opinion. Polit Behav. 2013;36(2):235–62. doi: 10.1007/s11109-013-9238-0

[11] Taber CS, Lodge M. Motivated Skepticism in the Evaluation of Political Beliefs. American J Political Sci. 2006;50(3):755–69. doi: 10.1111/j.1540-5907.2006.00214.x

[12] Kahan DM, Braman D, Cohen GL, Gastil J, Slovic P. Who fears the HPV vaccine, who doesn’t, and why? an experimental study of the mechanisms of cultural cognition. Law Hum Behav. 2010;34(6):501–16. doi: 10.1007/s10979-009-9201-0 20076997

[13] Kahan DM, Braman D. Cultural cognition and public policy. Yale L. & Pol’y Rev. 2006;24:149.

[14] Lu H, Chu H, Ma Y. Experience, experts, statistics, or just science? Predictors and consequences of reliance on different evidence types during the COVID-19 infodemic. Public Underst Sci. 2021;30(5):515–34. doi: 10.1177/09636625211009685 33892612

[15] Griffin RJ, Yang Z, Ter Huurne E, Boerner F, Ortiz S, Dunwoody S. After the flood: Anger, attribution, and the seeking of information. Sci Commun. 2008;29(3):285–315.

[16] Griffin RJ, Dunwoody S, Neuwirth K. Proposed model of the relationship of risk information seeking and processing to the development of preventive behaviors. Environ Res. 1999;80(2 Pt 2):S230–45. doi: 10.1006/enrs.1998.3940 10092438

[17] Hornikx J. A review of experimental research on the relative persuasiveness of anecdotal, statistical, causal, and expert evidence. Studies in Communication Sciences. 2005;5(1):205–16.

[18] Hinnant A, Hu S, Hong Y, Young R. Contested Certainty and Credibility: The Effect of Personal Stories and Scientific Evidence in User Comments on News Story Evaluation and Relevance. Science Communication. 2023;45(1):65–94. doi: 10.1177/10755470221150503

[19] Hinnant A, Subramanian R, Young R. User comments on climate stories: impacts of anecdotal vs. scientific evidence. Climatic Change. 2016;138:411–24.

[20] Justwan F, Baumgaertner B. The effects of ideology and cognitive reflection on evidence gathering behavior in the political domain. PLoS One. 2025;20(12):e0338088. doi: 10.1371/journal.pone.0338088 41329701 PMC12671747

[21] Christensen D. Higher-order evidence 1. Philosophy and Phenomenological Research. 2010;81(1):185–215.

[22] Tal E. Is higher-order evidence evidence?. Philos Stud. 2020;178(10):3157–75. doi: 10.1007/s11098-020-01574-0

[23] Wasserman EA, Dorner WW, Kao SF. Contributions of specific cell information to judgments of interevent contingency. J Exp Psychol Learn Mem Cogn. 1990;16(3):509–21. doi: 10.1037//0278-7393.16.3.509 2140406

[24] Kahan DM, Peters E, Dawson EC, Slovic P. Motivated numeracy and enlightened self-government. Behav Public Policy. 2017;1(1):54–86. doi: 10.1017/bpp.2016.2

[25] Levy N. Bad beliefs: Why they happen to good people. Oxford University Press. 2021. 35167201

[26] Cooper A, Doyle O, Bourke A. Supervised Clustering for Subgroup Discovery: An Application to COVID-19 Symptomatology. Communications in Computer and Information Science. Springer International Publishing. 2021. 408–22. 10.1007/978-3-030-93733-1_29

[27] Lundberg SM, Erion GG, Lee S. Consistent individualized feature attribution for tree ensembles. 2019.

[28] Al-Harbi SH, Rayward-Smith VJ. Adapting k-means for supervised clustering. Appl Intell. 2006;24(3):219–26. doi: 10.1007/s10489-006-8513-8

[29] Nohara Y, Matsumoto K, Soejima H, Nakashima N. Explanation of machine learning models using shapley additive explanation and application for real data in hospital. Comput Methods Programs Biomed. 2022;214:106584. doi: 10.1016/j.cmpb.2021.106584 34942412

[30] Lundberg SM, Lee S. A unified approach to interpreting model predictions. Advances in Neural Information Processing Systems. 2017;30.

[31] Cohen J, Huan X, Ni J. Shapley-based explainable AI for clustering applications in fault diagnosis and prognosis. J Intell Manuf. 2024;35(8):4071–86. doi: 10.1007/s10845-024-02468-2

[32] Byrd N. A two-factor explication of “reflection”: Unifying, making sense of, and guiding the philosophy and science of reflective reasoning. Res Philosophica. 2025;102(3):373–92.

[33] Pennycook G, Rand DG. Lazy, not biased: Susceptibility to partisan fake news is better explained by lack of reasoning than by motivated reasoning. Cognition. 2019;188:39–50. doi: 10.1016/j.cognition.2018.06.011 29935897

[34] Tullett AM, Hart WP, Feinberg M, Fetterman ZJ, Gottlieb S. Is ideology the enemy of inquiry? Examining the link between political orientation and lack of interest in novel data. Journal of Research in Personality. 2016;63:123–32. doi: 10.1016/j.jrp.2016.06.018

[35] Toplak ME, West RF, Stanovich KE. Assessing miserly information processing: An expansion of the Cognitive Reflection Test. Thinking & Reasoning. 2013;20(2):147–68. doi: 10.1080/13546783.2013.844729

[36] Brañas-Garza P, García-Muñoz T, González RH. Cognitive effort in the Beauty Contest Game. Journal of Economic Behavior & Organization. 2012;83(2):254–60. doi: 10.1016/j.jebo.2012.05.018

[37] Shook NJ, Fazio RH. Political ideology, exploration of novel stimuli, and attitude formation. Journal of Experimental Social Psychology. 2009;45(4):995–8. doi: 10.1016/j.jesp.2009.04.003

[38] Oechssler J, Roider A, Schmitz PW. Cognitive abilities and behavioral biases. Journal of Economic Behavior & Organization. 2009;72(1):147–52. doi: 10.1016/j.jebo.2009.04.018

[39] Jost JT, Napier JL, Thorisdottir H, Gosling SD, Palfai TP, Ostafin B. Are needs to manage uncertainty and threat associated with political conservatism or ideological extremity?. Pers Soc Psychol Bull. 2007;33(7):989–1007. doi: 10.1177/0146167207301028 17620621

[40] Frederick S. Cognitive Reflection and Decision Making. Journal of Economic Perspectives. 2005;19(4):25–42. doi: 10.1257/089533005775196732

[41] Jost JT, Glaser J, Kruglanski AW, Sulloway FJ. Political conservatism as motivated social cognition. Psychol Bull. 2003;129(3):339–75. doi: 10.1037/0033-2909.129.3.339 12784934

[42] Qualtrics. The XM Platform. 2024.

[43] Prolific. Quickly find research participants you can trust. 2024.

[44] Petty RE, Krosnick JA. Attitude strength: Antecedents and consequences. Petty RE, Krosnick JA. Lawrence Erlbaum Associates. 1–24. 1995.

[45] Griffin D, Tversky A. The weighing of evidence and the determinants of confidence. Cognitive Psychology. 1992;24(3):411–35. doi: 10.1016/0010-0285(92)90013-r

[46] Hastie TJ. Generalized Additive Models. In: Hastie TJ. Statistical Models in S. Routledge. 1992. 249–307.

[47] Harrell JFE. Ordinal logistic regression. Regression modeling strategies: with applications to linear models, logistic and ordinal regression, and survival analysis. 2015. 311–25.

[48] Kahl F, Kahl I, Jonas SM. XGBOrdinal: An XGBoost extension for ordinal data. Intelligent health systems–from technology to data and knowledge. IOS Press. 2025. 462–6. 10.3233/SHTI250380 40380490

[49] Van Rossum G, Drake FL. Python tutorial. Amsterdam, The Netherlands: Centrum voor Wiskunde en Informatica. 1995.

[50] Murtagh F, Legendre P. Ward’s Hierarchical Agglomerative Clustering Method: Which Algorithms Implement Ward’s Criterion?. J Classif. 2014;31(3):274–95. doi: 10.1007/s00357-014-9161-z

[51] R Core Team. R: A Language and Environment for Statistical Computing. 2025.

[52] Karlan B. Reasoning with heuristics. Ratio. 2020;34(2):100–8. doi: 10.1111/rati.12291

[53] Metzger MJ, Flanagin AJ. Credibility and trust of information in online environments: The use of cognitive heuristics. Journal of Pragmatics. 2013;59:210–20. doi: 10.1016/j.pragma.2013.07.012

[54] Strudler A, Warren DE. Authority, heuristics, and the structure of excuses. The Next Phase of Business Ethics: Integrating Psychology and Ethics. Emerald Group Publishing Limited. 2001. 355–75.

[55] Hjellbrekke J. Multiple correspondence analysis for the social sciences. Routledge. 2018.

[56] Maugeri A, Barchitta M, Basile G, Agodi A. Applying a hierarchical clustering on principal components approach to identify different patterns of the SARS-CoV-2 epidemic across Italian regions. Sci Rep. 2021;11(1):7082. doi: 10.1038/s41598-021-86703-3 33782519 PMC8007710

[57] Meyer A, Attali Y, Bar-Hillel M, Frederick S, Kahneman D. Cognitive reflection is a distinct and measurable trait. Proc Natl Acad Sci U S A. 2024;121(49):e2409191121. doi: 10.1073/pnas.2409191121 39602272 PMC11626181

[58] Liberali JM, Reyna VF, Furlan S, Stein LM, Pardo ST. Individual Differences in Numeracy and Cognitive Reflection, with Implications for Biases and Fallacies in Probability Judgment. J Behav Decis Mak. 2012;25(4):361–81. doi: 10.1002/bdm.752 23878413 PMC3716015

[59] Toplak ME, West RF, Stanovich KE. The Cognitive Reflection Test as a predictor of performance on heuristics-and-biases tasks. Mem Cognit. 2011;39(7):1275–89. doi: 10.3758/s13421-011-0104-1 21541821

[60] Ford N, Wilson TD, Foster A, Ellis D, Spink A. Information seeking and mediated searching. Part 4. Cognitive styles in information seeking. J Am Soc Inf Sci. 2002;53(9):728–35. doi: 10.1002/asi.10084

[61] Martín M, Valiña MD. Heuristics, Biases and the Psychology of Reasoning: State of the Art. PSYCH. 2023;14(02):264–94. doi: 10.4236/psych.2023.142016

[62] Deppe KD, Gonzalez FJ, Neiman JL, Jacobs C, Pahlke J, Smith KB, et al. Reflective liberals and intuitive conservatives: A look at the Cognitive Reflection Test and ideology. Judgment and Decision Making. 2015;10(4):314–31.

[63] Yilmaz O, Saribay SA. An attempt to clarify the link between cognitive style and political ideology: A non-western replication and extension. Judgment and Decision Making. 2016;11(3):287–300.

[64] Howe PDL, Perfors A, Walker B, Kashima Y, Fay N. Base rate neglect and conservatism in probabilistic reasoning: Insights from eliciting full distributions. Judgm decis mak. 2022;17(5):962–87. doi: 10.1017/s1930297500009281

[65] Keller PA, Lehmann DR. Designing Effective Health Communications: A Meta-Analysis. Journal of Public Policy & Marketing. 2008;27(2):117–30. doi: 10.1509/jppm.27.2.117

[66] Kreuter MW, Wray RJ. Tailored and targeted health communication: strategies for enhancing information relevance. Am J Health Behav. 2003;27 Suppl 3:S227-32. doi: 10.5993/ajhb.27.1.s3.6 14672383

[67] Morens DM, Fauci AS. Emerging Pandemic Diseases: How We Got to COVID-19. Cell. 2020;182(5):1077–92. doi: 10.1016/j.cell.2020.08.021 32846157 PMC7428724

[68] Sellier A-L, Scopelliti I, Morewedge CK. Debiasing Training Improves Decision Making in the Field. Psychol Sci. 2019;30(9):1371–9. doi: 10.1177/0956797619861429 31347444

[69] Akl EA, Oxman AD, Herrin J, Vist GE, Terrenato I, Sperati F, et al. Using alternative statistical formats for presenting risks and risk reductions. Cochrane Database Syst Rev. 2011;2011(3):CD006776. doi: 10.1002/14651858.CD006776.pub2 21412897 PMC6464912

[70] Hoffrage U, Lindsey S, Hertwig R, Gigerenzer G. Communicating statistical information. Science. 2000;290(5500):2261–2. 11188724 10.1126/science.290.5500.2261

[71] Gigerenzer G, Hoffrage U. How to improve Bayesian reasoning without instruction: Frequency formats. Psychological Review. 1995;102(4):684–704. doi: 10.1037/0033-295x.102.4.684

[72] Azzopardi L. Cognitive biases in search: a review and reflection of cognitive biases in information retrieval. In: Proceedings of the 2021 conference on human information interaction and retrieval, 2021. 27–37.

[73] Kim HK, Ahn J, Atkinson L, Kahlor LA. Effects of COVID-19 Misinformation on Information Seeking, Avoidance, and Processing: A Multicountry Comparative Study. Sci Commun. 2020;42(5):586–615. doi: 10.1177/1075547020959670 38603002 PMC7492825

[74] Byrd N, Joseph B, Gongora G, Sirota M. Tell Us What You Really Think: A Think Aloud Protocol Analysis of the Verbal Cognitive Reflection Test. J Intell. 2023;11(4):76. doi: 10.3390/jintelligence11040076 37103261 PMC10146599

[75] Purcell ZA, Howarth S, Wastell CA, Roberts AJ, Sweller N. Eye tracking and the cognitive reflection test: Evidence for intuitive correct responding and uncertain heuristic responding. Mem Cognit. 2022;50(2):348–65. doi: 10.3758/s13421-021-01224-8 34389912

[76] Cullen S, Byrd N, Chapkovski P, Thomason N. Thinking alone, and together: Dissenting pairs corrected more faulty decisions than solitary reasoners across four tasks. 2022.

