# Supplementary material for: Patterns between evidence seeking behaviors, reasoning, and cognitive reflection: A supervised clustering approach
Source: PLoS One. 2026 Jun 25;21(6):e0352096. doi: 10.1371/journal.pone.0352096 (PMC13298786; doi:10.1371/journal.pone.0352096)
Supplement: S1 Appendix — The appendix provides further information on research design, statistical analysis and results. This includes 9 Tables and 9 Figs. (PDF) [file pone.0352096.s001.pdf]

Supplementary Material for the paper "Patterns between evidence seeking behaviors, reasoning, and cognitive reflection: a supervised clustering approach"

Chénangnon Fréderic Tovissodé[1], Florian Justwan[1,2] and Bert Baumgaertner[1,2]

Contents

1 Supplement to methods **2**
1.1 Study design . . . 2
1.2 SHAP value calculation . . . 2
1.3 Cluster analysis for pattern identification . . . 3
1.4 Multinomial logistic regression . . . 7
1.5 Binary logistic regression . . . 7
1.6 Softwares and packages used . . . 9

2 Supplement to results **10**
2.1 Summaries of relevance of evidence and assessments . . . 10
2.2 Explaining assessment using gathered evidence . . . 11
2.3 Patterns in evidence gathering . . . 13
2.4 Demographic determinants of group memberships . . . 17

[1]Institute for Modeling Collaboration and Innovation (University of Idaho), USA
[2]Department of Politics and Philosophy (University of Idaho), USA

Corresponding author:
Chénangnon Fréderic Tovissodé, Institute for Modeling Collaboration and Innovation, University of Idaho, 875 Perimeter Drive, MS 3165, Moscow, ID 83844-3165, USA.
Email: ctovissode@uidaho.edu

1 Supplement to methods

This section gives additional details on the study methodology.

1.1 Study design

Our sample was designed to match the known population distribution in the U.S. on the dimensions of sex, age, and political affiliation. Table I shows that this goal was largely achieved. Our sample matches the U.S. population breakdown quite well in terms of Age, Gender, and Republican partisan affiliation. By contrast, Democrats are slightly over-represented in our sample (33% vs. 28%) whereas Independent are slightly under-represented (38% vs. 43%).

Table I. Demographic characteristics (Age, Gender and Political Affiliation) of U.S. population versus recruited participants

Variable	Population data (%)	Sample statistics (%)
Age group	*Census 2020*[1]	
18-24	12.10	12.36
25-34	17.35	18.14
35-44	16.33	17.73
45-54	15.95	15.76
55 or older	37.63	36.01
Gender	*Census 2020*[1]	
Woman	50.93	51.21
Man	49.07	48.79
Political Affiliation	*Gallup 2024*[2]	
Republican	28.00	28.25
Democrat	28.00	33.36
Independent	43.00	38.39

1.2 SHAP value calculation

We detail here the SHAP methodology. The SHAP value is the amount that a particular piece of evidence (ev) contributes to the prediction of an individual's assessment Z beyond a baseline average prediction. Given the prediction model f (our XGBoost tree machine), the SHAP value, denoted $\phi_j(f, x)$, of a piece of evidence ev_j ($j = 1 : 12$) for a respondent x is collectively defined for all ev through (1)[3]:

$$f(x) = \phi_0(f) + \sum_{j=1}^{p} \phi_j(f, x) \tag{1}$$

where $\phi_0(f)$ is the baseline average predicted assessment Z by the model f, and $p = 12$ is the total number of available ev. By expressing the target prediction from the nonlinear

predictive model f as a linear combination of the baseline prediction $\phi_0(f)$ and the selection or not of available ev_j, (1) highlights the interpretability of SHAP value as marginal additive contributions of predictors to the local expected value $f(x)$. Intuitively, if $\phi_j(f, x)$ is positive, the selection of ev_j by the respondent x would increase the predicted assessment $f(x)$. The non-selection of ev_j would conversely reduce $f(x)$. For a negative $\phi_j(f, x)$, the selection of ev_j would reduce $f(x)$ while the non-selection of ev_j would increase $f(x)$.

For explicit computations, the mathematical formula for the SHAP value is given by (2)[4]:

$$\phi_j(f, x) = \sum_{ev_s \subset E \setminus \{ev_j\}} \frac{|ev_s|!(p - |ev_s| - 1)!}{p!} \left[f_x \left(ev_s \bigcup ev_j \right) - f_x(ev_s) \right] \tag{2}$$

where E is the power set of all ev, ev_s is a piece of evidence subset that excludes ev_j, $f_x\left(ev_s \bigcup ev_j\right) - f_x(ev_s)$ is the marginal contribution of adding ev_j to the evidence subset ev_s. (2) shows that each SHAP value is a weighted average of differences over all possible subsets ev_s. In accordance with (2), the calculation of each SHAP value $\phi_j(f, x)$ is a computationally demanding task since it requires repeated evaluations of f. In our setting, the set E includes $2^{12} = 4096$ elements for each of our 2909 included respondents. The SHAP values described in this paper were obtained using Lundberg et al.[5]'s approximation as implemented by the package *shap*[6] in the Python[7] programming language.

1.3 Cluster analysis for pattern identification

We considered cluster analysis to identify patterns in evidence gathering (considering sources of evidence respondents selected when they were presented with the 12 pieces of evidence). Hierarchical Clustering (HC) allowed us to explore relationships between clusters, in both initial and final evidence gathering data. In order to build the most compact and well separated clusters of respondents, we compared two different approaches to clustering with the goal to identify the best one: unsupervised clustering which starts with the original binary data, and supervised clustering which uses SHAP values obtained from an XGBoost machine.

For the first approach, unsupervised clustering, since the evidence gathering data is binary, we computed the Hamming distance[8] on a 12-column binary matrix representing pieces of evidence selected by participants. Given two respondents, the Hamming distance is the number of pieces of evidence differentially selected (one respondent selected and the other did not select). On the Hamming distance matrix, we ran HC to obtain a nested hierarchy among potential groups of respondents. We then took advantage of dimension reduction techniques to visualize the tree of hierarchy among potential groups in the first few dimensions retaining the statistically most relevant information in the data[9]. Specifically, we performed a Principal Coordinates Analysis (PCoA)[10] to obtain a representation of the HC result.

The second approach, supervised clustering, is similar to classification and regression in that, a target response variable is required. However, instead of predicting the classes or

values of the response variable, the aim is to deliver a clustering in which each cluster has a strong tendency to have the same class or close values of the response [11]. In this case, we ran both HC and PCA on SHAP values so as to visualize the hierarchy of potential clusters from the HC using the main information extracted from SHAP values by PCA.

To access the internal cohesion of clusters, we computed a measure of compactness and separation of clusters, namely the silhouette score [12,13], varying the number of clusters in the range $[2, 6]$. The Silhouette value measures the degree of confidence in a particular clustering assignment and lies in the interval $[-1, 1]$, with well-clustered observations having values near 1 and poorly clustered observations having values near -1 (the larger the better). We also computed the average proportion of non-overlap (APN) [14] as stability (consistency) measure. The APN measures the average proportion of observations not placed in the same cluster after deletion of one input data column. Indeed, the APN evaluates the stability of a clustering result by comparing it with the clusters obtained by removing one data column at a time. The average is taken over all the deleted columns, and the lower the APN value, the better.

For comparison purposes, we computed these two indices for clusters derived from unsupervised and supervised clustering methods. Table II displays cluster validation measures for supervised and unsupervised hierarchical clustering methods. It appears that supervised clustering provides the most well separated (higher silhouette score) and most stable clusters (lower APN) for all considered k values. As illustration, Fig I shows the tree of hierarchy among groups of respondents (resulting from HC) in the principal plane accounting for 43% of the variability in evidence data. It appears difficult to visually delineate frontiers between groups of respondents, even considering only two groups. In comparison, supervised clustering leads to clear separation between groups up to five groups of respondents (Fig 1 and VII). Finally note that for the supervised clustering method (Table II), partitioning the data into $k = 3$ clusters provides the most well separated clusters ($Silhouette\ =\ 0.57$), but $k = 2$ leads to most stable clusters ($APN\ =\ 0.16$).

The difference between unsupervised clustering and supervised clustering in terms of information content is the assessment of the effectiveness (Z) of the nasal spray by respondents. However, supervised clustering discards all evidence information not useful for predicting Z. This is different from simply feeding all evidence data and Z to an unsupervised clustering algorithm. Indeed, Table III compares unsupervised hierarchical clustering on multiple correspondence analysis, using only the selected evidence data, versus using a combination of evidence data and Z. Both these approaches produce less well separated and stable clusters. In addition, including Z in unsupervised clustering actually results in less well separated and less stable clusters (Table III).

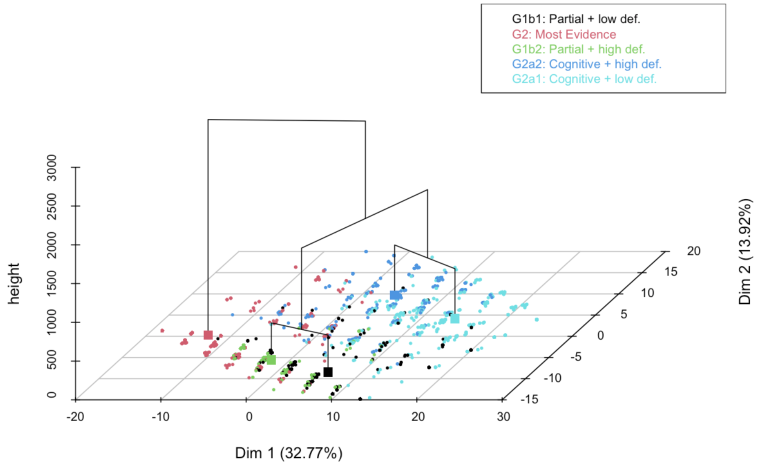

Fig I. Results of Hierarchical Clustering (HC) showing the tree of hierarchy and five clusters of respondents ($N = 2909$) in the first two Principal Components (PC). An HC and a Principal Coordinates Analysis (PCoA) were performed on the 12 pieces of evidence selected by respondents. Each dot on the graphic represents a folk and colors indicate group memberships. The indicated labels of clusters were derived from the characterization of the five clusters based on the tree of hierarchy and the proportion of respondents who selected different pieces of evidence in each cluster.

Table II. Cluster validation measures for a number of clusters k (in the range $[2, 6]$) derived from unsupervised clustering (using binary evidence data) and supervised clustering (using SHAP values based on predicting the assessment of the effectiveness of nasal spray given the total selected evidence).

**Measure**	**k - unsupervised clustering**					**k - supervised clustering**				
	2	3	4	5	6	2	3	4	5	6
Silhouette	0.28	0.27	0.27	0.22	0.20	0.56	0.57	0.54	0.53	0.52
APN	0.36	0.31	0.40	0.44	0.46	0.16	0.20	0.26	0.29	0.32

Silhouette = the degree of confidence in a particular clustering assignment and lies in the interval $[-1, 1]$, with well-clustered observations having values near 1 and poorly clustered observations having values near -1; *APN* = for two partitions, the average proportion of observations not placed in the same cluster under the partition and a second partition based on the same data with one column deleted, average over all the deleted columns;

Table III. Cluster validation measures for a number of clusters k (in the range $[2, 6]$) derived from unsupervised Hierarchical Clustering on Multiple Correspondence Analysis (HCMCA) using either only the selected evidence data, or the selected evidence data and respondents' assessments of the effectiveness of nasal spray given the total selected evidence (Z).

**Measure**	**k (HCMCA on evidence)**					**k (HCMCA on evidence and Z)**				
	2	3	4	5	6	2	3	4	5	6
Silhouette	0.15	0.16	0.18	0.18	0.15	0.11	0.08	0.09	0.11	0.15
APN	0.04	0.15	0.21	0.23	0.28	0.33	0.48	0.36	0.31	0.30

Silhouette = the degree of confidence in a particular clustering assignment and lies in the interval $[-1, 1]$, with well-clustered observations having values near 1 and poorly clustered observations having values near -1; *APN* = for two partitions, the average proportion of observations not placed in the same cluster under the partition and a second partition based on the same data with one column deleted, average over all the deleted columns;

1.4 Multinomial logistic regression

To test the statistical hypothesis H_1 in the main text, we fitted a Multinomial Logistic Regression (MNLR) model to group membership against CRT-7 total score, and ideology. We considered demographic characteristics such as gender (man or not), age, race (white or not), and education level as potential confounding variables. We used a backward model selection approach to eliminate confounding variables with non-significant contributions to the model fit. The dataset shows overall a 5.7% Percentage of Respondents who were Partial Respondents (PRPR). Given such a low PRPR ($< 10\%$), we reported MNLR results based on a pairwise deletion of missing values following Newman[15]'s guidelines. We checked the goodness-of-fit of the final model using the Hosmer and Lemeshow test[16] (see Table IX).

To investigate the robustness of our results to the presence of missing data, we tested if data were missing completely at random (MCAR) using Little[17]'s MCAR test. The test result ($\chi^2_8 = 9.84$, $P = 0.277$) indicates that data were MCAR. In accordance with this result, we used multiple imputation with $m = 100$ data replicates to check the robustness of the reported MNLR fit results to missingness. We pooled χ^2 statistics from the m individual MNLR fits into Fisher statistics following[18].

Tables IV and V present multiple imputation based MNLR fit results, analogous to the MNLR fit results in the section *Demographic determinants of group memberships* of the main text. The pooled Hosmer and Lemeshow test indicates a good fit ($F_{(32.00,\ 2755.55)} = 0.731$, $P = 0.8652$). The pooled MNLR results indicate that our findings are robust to the observed level of missingness. Indeed, the analysis of deviance Table IV shows that CRT-7 total score ($F_{(4.00,18154.64)} = 20.00$, $P < 0.001$) significantly affects group membership while Ideology ($F_{(4.00,\ 45986.36)} = 1.65$, $P = 0.158$) is not significantly associated with group membership. Table V presents model terms and pooled coefficients and odd ratios.

Table IV. Pooled analysis of deviance results on the multinomial logistic regression fit to group membership against CRT-7 total score, ideology, gender, and age of respondents. $F_{(\nu_1,\nu_2)}$ is the Fisher statistic with degrees of freedom ν_1 and ν_2. The $F_{(\nu_1,\nu_2)}$ statistics resulted from pooling χ^2 statistics following[18]. Pooled Hosmer and Lemeshow goodness-of-fit test: $F_{(32.00,\ 2755.55)} = 0.731$, $P = 0.8652$.

Predictor	ν_1	ν_2	$F_{(\nu_1,\nu_2)}$	P
CRT-7 total score	4	18154.640	19.995	<0.0001
Ideology	4	45986.360	1.652	0.1582
Gender (Man=1)	4	2015327.000	1.502	0.1986
Age	4	24442230.000	2.997	0.0174

1.5 Binary logistic regression

An alternative approach to MNLR for testing H_1 is the Binary Logistic Regression (BLR) fitted to the selection or not of the full first-order evidence (ev_1-ev_4) against

Table V. Pooled multinomial logistic regression fit results (pooled Hosmer and Lemeshow goodness-of-fit test: $F_{32,\ 2755.55} = 0.731$, $P = 0.8652$).

Group	Model term	Estimate	Odd ratio*	$CI_{95\%}$**
G1b1: Partial + low def.	(Intercept)	1.810	-	-
	CRT-7 total score	0.068	1.070	[0.960, 1.193]
	Ideology	0.113	1.120	[0.917, 1.367]
	Gender (Man=1)	-0.281	0.755	[0.482, 1.183]
	Age	-0.025	0.976	[0.849, 1.121]
G1b2: Partial + high def.	(Intercept)	-0.163	-	-
	CRT-7 total score	0.113	1.120	[0.972, 1.290]
	Ideology	0.084	1.087	[0.834, 1.417]
	Gender (Man=1)	-0.159	0.853	[0.467, 1.557]
	Age	-0.085	0.919	[0.763, 1.105]
G2a: Full + def.	(Intercept)	2.270	-	-
	CRT-7 total score	0.210	1.234	[1.110, 1.372]
	Ideology	0.055	1.057	[0.869, 1.286]
	Gender (Man=1)	-0.348	0.706	[0.455, 1.095]
	Age	-0.037	0.963	[0.841, 1.103]
G2b: Most Evidence	(Intercept)	1.860	-	-
	CRT-7 total score	0.245	1.278	[1.145, 1.426]
	Ideology	-0.007	0.993	[0.810, 1.218]
	Gender (Man=1)	-0.456	0.634	[0.401, 1.003]
	Age	-0.130	0.878	[0.763, 1.011]

* Odd ratios are given with reference to the group "G1a: Scant evidence".
** $CI_{95\%}$ is the 95% confidence interval for the population odd ratio.

the same predictors. More that half of respondents (58%, 1684/2909) selected full first-order data. For this BLR analysis, our stepwise model selection excluded all potential confounding variables, retaining only the CRT-7 total score as explanatory variable. The result is consistent with the MNLR result (Table VI): the probability to seek full first-order data significantly increased with the CRT-7 total score of respondents ($\chi^2_1 = 12.80$, $P < 0.001$).

Table VI. Binomial logistic regression fit to the selection or not of full first order data against CRT-7 total score ($N = 2909$). Hosmer and Lemeshow goodness-of-fit test: $\chi^2_8 = 12.480$, $P = 0.1312$.

Model term	Estimate	Odd ratio	$CI_{95\%}$**
(Intercept)	-2.019	-	-
CRT-7 total score	0.12	1.130	[1.060, 1.210]

** $CI_{95\%}$ is the 95% confidence interval for the population odd ratio.

1.6 Softwares and packages used

We used the packages *xgboost*[19] for XGBoost machine learning and *shap*[6] for the computation of SHAP values in the Python (version 3.10.13) programming language[7] along with *sklearn*[20] for linear model, *mord*[21] for ordinal regression, *pygam* for generalized additive model. All other analyses were performed in the R (version 4.5.1) statistical environment[22] and the significance level was set to 5% for statistical tests. We used the R packages *vegan*[23] to compute distances for HC, *FactoMineR*[24] for PCA, CA and MCA, *clValid*[25] for cluster validation measures, *nnet*[26] to fit MNLR models, *naniar*[27] to perform Little's MCAR test, *mice*[28] for multiple imputation, *miceadds*[29] to pool χ^2 statistics from imputed datasets, *generalhoslem*[30] to perform Hosmer and Lemeshow test, *ggplot2*[31] and *pheatmap*[32] for visualizations of cluster descriptive statistics. All data and codes used for analyses are available via OSF at https://osf.io/78kxp/?view_only=fd5aafd8a4ab4e1ca6cf679b3164bccb.

2 Supplement to results

This section presents additional details on results related to demographic characteristics of respondents, the predictive modeling of respondents' assessment of the effectiveness of the nasal spray against COVID-19, the identification of patterns in respondents' selection of proposed evidence, and how these patterns relate to demographic factors.

2.1 Summaries of relevance of evidence and assessments

The percentage of respondents who selected each of the 12 pieces of evidence is shown on Fig II. We observed that most pieces of evidence (9/12) were selected by more than half of respondents, with ev_1 = "employee-use-infected" being requested by 94% of folks. Evidence selected by only a minority include ev_8 = "company-name" (38% of folks), ev_{11} = "animal-studies" (25%) and ev_{12} = "india-health-authorities" (24%). About 6% of folks (179/2951) selected all 12 pieces of evidence at the first round. In fact most folks (54%, 1594/2951) selected only six or less of the 12 pieces of evidence as sources of primary relevance. Moreover, only 25% (i.e. 683/2772) of folks who could have selected more evidence have taken the opportunity to gather more evidence. Indeed, each evidence was of secondary relevance very for few respondents ($\leq 8\%$, Fig II).

The assessment of the effectiveness of the nasal spray by respondents was on average $\bar{Z} = -0.76$ with a standard deviation of 2.31. The median value is $Z = -2$. Fig III shows that $Z = -2$ and $Z = -3$ (somewhat/fairly certain the the spray does NOT reduce infection rate) are the most frequent assessments of the effectiveness of the nasal spray among respondents.

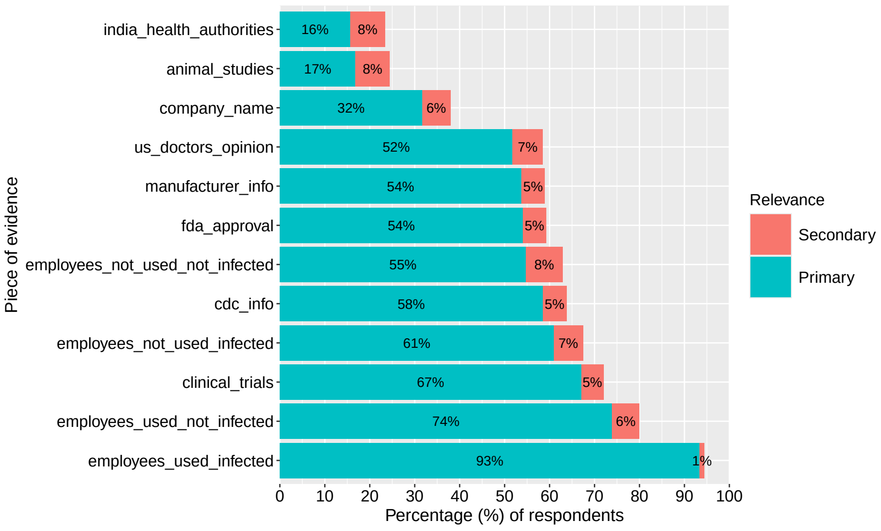

Fig II. Percentage of folks ($N = 2951$) who selected different pieces of evidence presented to respondents. "Primary" refers to evidence selected during the first round of selection, and "Secondary" refers to evidence selected when given the opportunity to gather additional evidence.

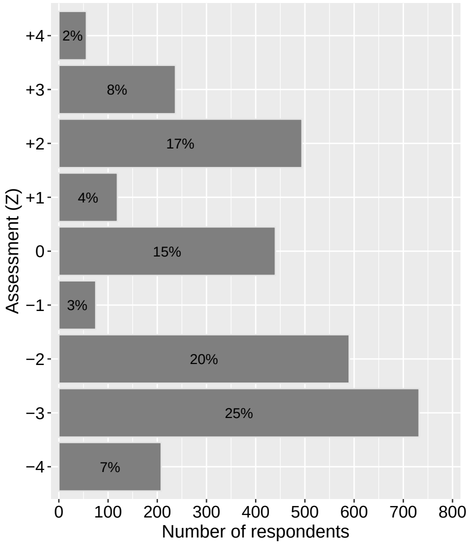

Fig III. Distribution of the assessments (Z) of the effectiveness of the nasal spray among all respondents ($N = 2951$). The percentages on the barplots show the proportion of respondents corresponding to each of Z value.

2.2 Explaining assessment using gathered evidence

Fig IV shows the mean absolute SHapley Additive exPlanation values (SHAP) values for the 12 pieces of evidence, computed from the best predictive model for the response Z. Fig V shows the results of Correspondence Analysis on respondents' assessment of the nasal spray effect (and the related confidence) Z and the most discriminating pieces of evidence ev_3 = "employees-not-used-infected" and ev_{12} = "india-health-authorities".

Fig VI shows the 12 pieces of evidence in the first two dimensions from the Multiple Correspondence Analysis on the total evidence data. It appears that all evidence pieces are positively correlated, but we have two clusters of pieces of evidence: most first order evidence (ev_2, ev_3 and ev_4) on the one hand and other pieces of evidence on the other hand.

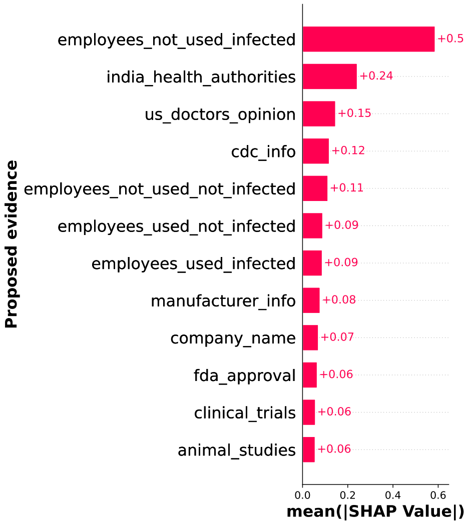

Fig IV. Mean absolute SHAP values for the 12 pieces of evidence explaining $N = 2909$ respondents's assessment of the effectiveness of the nasal spray against COVID-19 (including self-reported uncertainty or confidence in the response) based on the selected pieces of evidence. SHAP values were obtained from XGBoost machine trained on the whole data (after the model was selected by training and testing on respectively 70% and 30% of the data) and using the whole data as the background distribution of predictors.

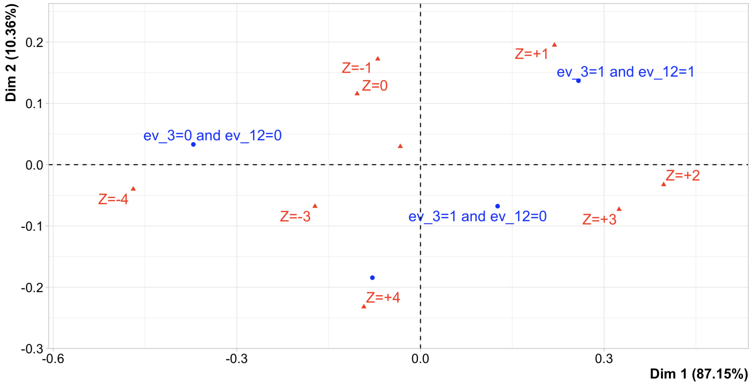

Fig V. Map of Correspondence Analysis on $N = 2909$ respondents' assessment of the nasal spray effect (and the related confidence) Z and the most discriminating pieces of evidence $ev_3 =$ "employees-not-used-infected" and $ev_{12} =$ "india-health-authorities". The first two axes retain 98% of the variability in the original data. Only well represented modalities (above average contributions, and square cosines above 0.5) are labeled. The not-well represented (and not labeled) modalities include "ev_3=0 and ev_12=1" (blue dot closest to $Z = +4$) and $Z = -2$ (red triangle closest to the origin). χ^2 test of independence between the assessment and the two pieces of evidence: $\chi^2_{24} = 204.36$, $P < 0.001$.

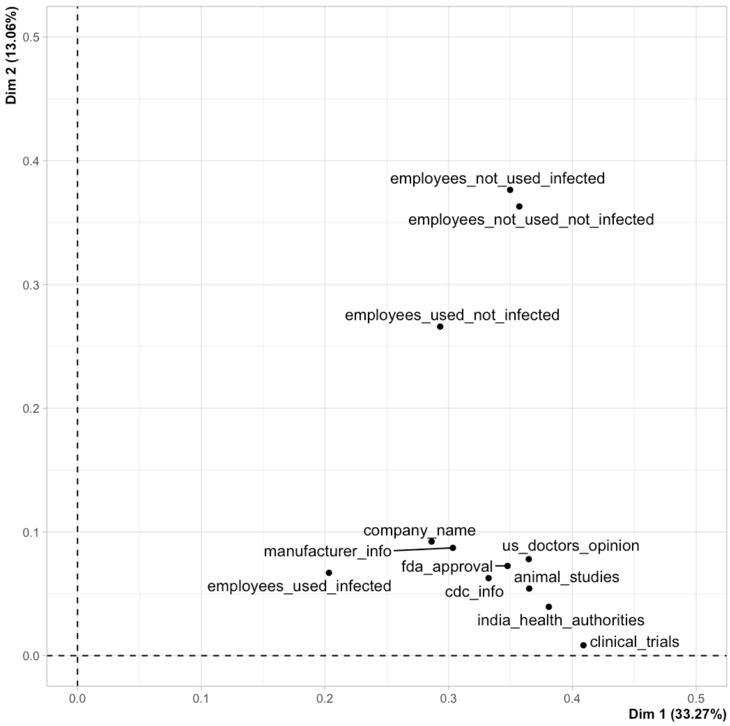

Fig VI. Map of Multiple Correspondence Analysis on $N = 2909$ respondents' choices to select or not select each of the proposed 12 pieces of evidence (see bank of evidence in Table 2 in the main text). The first two axes retain 46% of the variability in the original data.

2.3 Patterns in evidence gathering

Fig VII complements Fig 1 in the main text. Indeed, only four clusters are clearly separated in Fig 1 because the third Principal Component (PC) is not shown. Fig VII shows the tree of hierarchy among groups of respondents resulting from HC in the PC dimensions 1 and 3.

Fig VIII and IX show the correspondence between the five empirical groups and respondents' assessment of the effectiveness of the nasal spray (Fig VIII), and the CRT-7 total score (Fig IX). Table VII further gives the conditional percentages of different answers given the empirical group of an individual.

Table VII. Percentage of respondents who assessed the nasal spray as effective or ineffective, or were undecided about the effect, within each empirical group (the group labels are as defined in Fig 2).

**Empirical group**	**Ineffective** (%) $[CI_{95\%}]^*$	**Undecided** (%) $[CI_{95\%}]^*$	**Effective** (%) $[CI_{95\%}]^*$	**Total** (%)
G1a: Scant evidence	45.65 [35.22, 56.37]	17.39 [10.28, 26.70]	36.96 [27.12, 47.66]	100
G1b1: Partial + low def.	73.10 [69.74, 76.27]	13.18 [10.82, 15.84]	13.72 [11.32, 16.42]	100
G1b2: Partial + high def.	56.04 [45.25, 66.44]	14.29 [07.83, 23.19]	29.67 [20.55, 40.16]	100
G2a: Full + def.	51.15 [48.49, 53.81]	12.52 [10.82, 14.37]	36.33 [33.80, 38.92]	100
G2b: Most evidence	43.83 [39.82, 47.91]	16.33 [13.46, 19.54]	39.83 [35.89, 43.88]	100

* $CI_{95\%}$ is the 95% confidence interval for each percentage. The $CI_{95\%}$ are measures of variability and are not intended for comparisons of empirical groups because the assessment of the nasal spray (Ineffective, Undecided, Effective) was used as part of the supervised clustering methodology that resulted in the five groups (the definition of the row categories is, in part, based on the column categories hence the data are not independent).

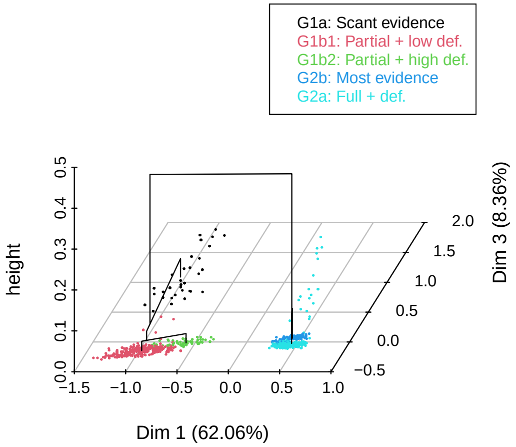

Fig VII. Results of Hierarchical Clustering (HC) showing the tree of hierarchy and five clusters of respondents in the first and third Principal Components (PC) dimensions. SHapley Additive exPlanation (SHAP) values were obtained from an XGBoost machine explaining $N = 2909$ folk's assessment of the effectiveness of the nasal spray against COVID-19 based on which of the 12 proposed pieces of evidence they selected. An HC and a PC analysis were then performed on the SHAP values of the 12 pieces of evidence. Each dot on the graphic represents a folk and colors indicate group memberships. The indicated labels of clusters were derived from the characterization of the five clusters based on the tree of hierarchy and the proportion of folks who selected different pieces of evidence in each cluster. The tree of hierarchy however shows that the group "G1b2: Partial + high def." (green) is closer to "G1b1: Partial + low def." (red) than "G1a: Scant evidence" (black) is to G1b1.

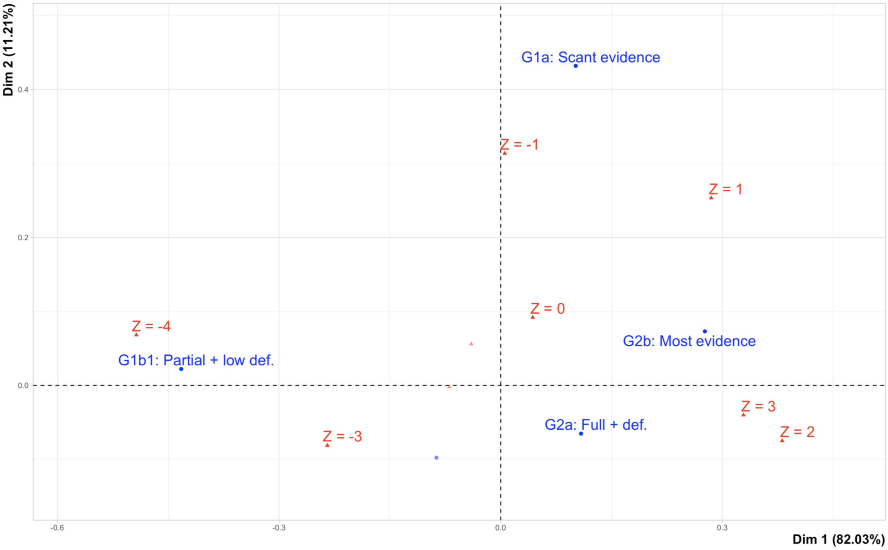

Fig VIII. Map of Correspondence Analysis on $N = 2909$ respondents' groups (blue dots) and assessments of the nasal spray effect (and the related confidence) Z (red triangles). The first two axes retain 93% of the variability in the original data. Only well represented modalities (above average contributions, and square cosines above 0.5) are labeled. The not-well represented (and not labeled) modalities include "G1b2: Partial + high def." (blue dot on the left of "G2a: Full + def."), and $Z = -2$ and $Z = 4$ (red triangles closest to the origin). χ^2 test of independence between the assessments and the groups: $\chi^2_{32} = 245.60,\ P < 0.001.$

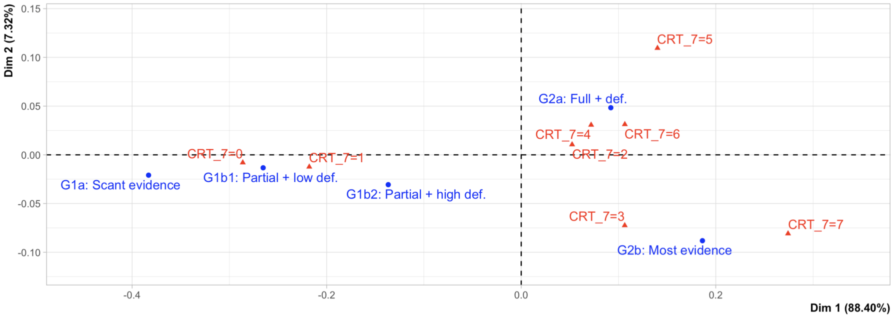

Fig IX. Map of Correspondence Analysis on $N = 2909$ respondents' groups (blue dots) and CRT-7 total scores (red triangles). The first two axes retain 96% of the variability in the original data. Only well represented modalities (above average contributions, and square cosines above 0.5) are labeled. The not-well represented modalities include "G1b2: Partial + high def." and CRT-7 = 2. Results of χ^2 test of independence between the assessments and the groups: $\chi^2_{32} = 105.98,\ P < 0.001.$

2.4 Demographic determinants of group memberships

Tables VIII and IX present the results of multinomial logistic regression fit to group membership against the CRT-7 total score, the ideology, the gender, and the age of respondents. The Hosmer and Lemeshow test indicates an overall good fit. The analysis of deviance Table VIII shows that CRT-7 total score ($\chi^2_4 = 81.04$, $P < 0.001$) significantly affects group membership while Ideology ($\chi^2_4 = 5.94$, $P = 0.204$) is not significantly associated with group membership. It also appears that respondents' age is significant confounder for group membership ($\chi^2_4 = 14.00$, $P = 0.007$): older respondents were more likely to belong to the group G1a than younger respondents. Table IX presents model terms and coefficients.

Table VIII. Analysis of deviance results on the multinomial logistic regression fit to group membership against CRT-7 total score, ideology, gender, and age of respondents ($N = 2909$). Hosmer and Lemeshow goodness-of-fit test: $\chi^2_{32} = 33.844$, $P = 0.3785$.

**Predictor**	**Number of degrees of freedom**	χ^2_{df}	P
CRT-7 total score	4	81.040	<0.0001
Ideology	4	5.936	0.2040
Gender	4	7.869	0.0965
Age	4	14.009	0.0073

Table IX. Multinomial logistic regression fit to group membership (from supervised clustering on SHAP values for respondents' assessment of the effectiveness of the nasal spray against COVID-19 given total evidence data) against CRT-7 total score, ideology, gender, and age of respondents ($N = 2909$). Hosmer and Lemeshow goodness-of-fit test: $\chi^2_{32} = 33.844$, $P = 0.3785$.

Group	Model term	Estimate	Odd ratio*	$CI_{95\%}$**
G1b1: Partial + low def.	(Intercept)	1.860	-	-
	CRT-7 total score	0.077	1.080	[0.967, 1.206]
	Ideology	0.120	1.127	[0.921, 1.380]
	Gender (Man = 1)	-0.374	0.688	[0.430, 1.100]
	Age	-0.028	0.972	[0.840, 1.125]
G1b2: Partial + high def.	(Intercept)	-0.101	-	-
	CRT-7 total score	0.126	1.134	[0.984, 1.309]
	Ideology	0.110	1.116	[0.853, 1.461]
	Gender (Man = 1)	-0.251	0.778	[0.417, 1.451]
	Age	-0.109	0.897	[0.740, 1.087]
G2a: Full + def.	(Intercept)	2.340	-	-
	CRT-7 total score	0.228	1.256	[1.129, 1.399]
	Ideology	0.062	1.064	[0.874, 1.297]
	Gender (Man = 1)	-0.470	0.625	[0.395, 0.989]
	Age	-0.048	0.954	[0.827, 1.099]
G2b: Most Evidence	(Intercept)	1.950	-	-
	CRT-7 total score	0.264	1.302	[1.165, 1.455]
	Ideology	0.003	1.003	[0.816, 1.231]
	Gender (Man = 1)	-0.581	0.559	[0.346, 0.901]
	Age	-0.147	0.863	[0.745, 1.001]

∗ Odd ratios are given with reference to the group "G1a: Scant evidence".
∗∗ $CI_{95\%}$ is the 95% confidence interval for the population odd ratio.

References

1. US Census Bureau. Age and sex: American community survey 5-year estimates subject tables, table s0101, 2022. URL https://data.census.gov/table/ACSST5Y2022.S0101?q=people. Accessed on July 1, 2024.
2. Gallup. Party affiliation, 2024. URL https://news.gallup.com/poll/15370/party-affiliation.aspx. Accessed on July 1, 2024.
3. Cohen J, Huan X and Ni J. Shapley-based explainable AI for clustering applications in fault diagnosis and prognosis. *Journal of Intelligent Manufacturing* 2024; 35(8): 4071–4086.
4. Chen H, Covert IC, Lundberg SM et al. Algorithms to estimate shapley value feature attributions. *Nature Machine Intelligence* 2023; 5(6): 590–601.
5. Lundberg SM and Lee SI. A unified approach to interpreting model predictions. *Advances in neural information processing systems* 2017; 30.
6. Lundberg SM, Nair B, Vavilala MS et al. Explainable machine-learning predictions for the prevention of hypoxaemia during surgery. *Nature Biomedical Engineering* 2018; 2(10): 749.

7. Van Rossum G and Drake Jr FL. *Python tutorial*. Centrum voor Wiskunde en Informatica Amsterdam, The Netherlands, 1995.
8. Hamming RW. Error detecting and error correcting codes. *The Bell system technical journal* 1950; 29(2): 147–160.
9. Maugeri A, Barchitta M, Basile G et al. Applying a hierarchical clustering on principal components approach to identify different patterns of the sars-cov-2 epidemic across italian regions. *Scientific reports* 2021; 11(1): 7082.
10. Gower JC. Some distance properties of latent root and vector methods used in multivariate analysis. *Biometrika* 1966; 53(3-4): 325–338.
11. Al-Harbi SH and Rayward-Smith VJ. Adapting k-means for supervised clustering. *Applied Intelligence* 2006; 24: 219–226.
12. Rousseeuw PJ. Silhouettes: a graphical aid to the interpretation and validation of cluster analysis. *Journal of Computational and Applied Mathematics* 1987; 20: 53–65.
13. Januzaj Y, Beqiri E and Luma A. Determining the optimal number of clusters using silhouette score as a data mining technique. *International Journal of Online & Biomedical Engineering* 2023; 19(4).
14. Wo J, Zhang C, Xu B et al. Performances of clustering methods considering data transformation and sample size: An evaluation with fisheries survey data. *Journal of Ocean University of China* 2020; 19: 659–668.
15. Newman DA. Missing data: Five practical guidelines. *Organizational research methods* 2014; 17(4): 372–411.
16. Fagerland MW and Hosmer DW. A generalized hosmer–lemeshow goodness-of-fit test for multinomial logistic regression models. *The Stata Journal* 2012; 12(3): 447–453.
17. Little RJ. A test of missing completely at random for multivariate data with missing values. *Journal of the American statistical Association* 1988; 83(404): 1198–1202.
18. Enders CK. *Applied missing data analysis*. Guilford Publications, 2010.
19. Chen T and Guestrin C. XGBoost: A scalable tree boosting system, 2016. DOI:10.1145/2939672.2939785. URL http://doi.acm.org/10.1145/2939672.2939785.
20. Pedregosa F, Varoquaux G, Gramfort A et al. Scikit-learn: Machine learning in Python. *Journal of Machine Learning Research* 2011; 12: 2825–2830.
21. Pedregosa F, Bach F and Gramfort A. On the consistency of ordinal regression methods. *Journal of Machine Learning Research* 2017; 18(55): 1–35.
22. R Core Team. *R: A Language and Environment for Statistical Computing*. R Foundation for Statistical Computing, Vienna, Austria, 2025. URL https://www.R-project.org/.
23. Oksanen J, Simpson GL, Blanchet FG et al. *vegan: Community Ecology Package*. R Foundation for Statistical Computing, 2025. URL https://CRAN.R-project.org/package=vegan. R package version 2.6-10.
24. Lê S, Josse J and Husson F. FactoMineR: A package for multivariate analysis. *Journal of Statistical Software* 2008; 25(1): 1–18. DOI:10.18637/jss.v025.i01.
25. Brock G, Pihur V, Datta S et al. clValid: An R package for cluster validation. *Journal of Statistical Software* 2008; 25(4): 1–22. URL https://www.jstatsoft.org/v25/i04/.

26. Venables WN and Ripley BD. *Modern Applied Statistics with S*. Fourth ed. New York: Springer, 2002. URL https://www.stats.ox.ac.uk/pub/MASS4/. ISBN 0-387-95457-0.
27. Tierney N and Cook D. Expanding tidy data principles to facilitate missing data exploration, visualization and assessment of imputations. *Journal of Statistical Software* 2023; 105(7): 1–31. DOI:10.18637/jss.v105.i07.
28. van Buuren S and Groothuis-Oudshoorn K. mice: Multivariate imputation by chained equations in r. *Journal of Statistical Software* 2011; 45(3): 1–67. DOI:10.18637/jss.v045.i03.
29. Robitzsch A and Grund S. *miceadds: Some Additional Multiple Imputation Functions, Especially for 'mice'*. R Foundation for Statistical Computing, 2024. URL https://CRAN.R-project.org/package=miceadds. R package version 3.17-44.
30. Jay M. *generalhoslem: Goodness of Fit Tests for Logistic Regression Models*. R Foundation for Statistical Computing, 2019. URL https://CRAN.R-project.org/package=generalhoslem. R package version 1.3.4.
31. Wickham H. *ggplot2: Elegant Graphics for Data Analysis*. Springer-Verlag New York, 2016. ISBN 978-3-319-24277-4. URL https://ggplot2.tidyverse.org.
32. Kolde R. *pheatmap: Pretty Heatmaps*. R Foundation for Statistical Computing, 2019. URL https://CRAN.R-project.org/package=pheatmap. R package version 1.0.12.
