# Supplementary material for: Patterns between evidence seeking behaviors, reasoning, and cognitive reflection: A supervised clustering approach
Source: PLoS One. 2026 Jun 25;21(6):e0352096. doi: 10.1371/journal.pone.0352096 (PMC13298786; doi:10.1371/journal.pone.0352096)
Supplement: Attachment — Submitted filename: Response to Reviewers.pdf [file pone.0352096.s007.pdf]

Dear Editor of Plos One,

I am submitting this letter in response to the decision: Revision required [PONE-D-25-61437] (by April 24, 2026).

Thank you for considering our submission and giving us the opportunity to revise our manuscript. As you required, we have added the following statement to the financial disclosure in the manuscript: “The funders had no role in study design, data collection and analysis, decision to publish, or preparation of the manuscript.” Moreover, after reviewing the publications suggested by the reviewers, we have cited those relevant to the revisions we made. We reorganized the introduction and included all the necessary information and references to understand and contextualize the research.

My co-authors and I are grateful to the two reviewers for the comments and constructive suggestions to improve both the content and the presentation of the manuscript. We thoroughly revised the manuscript accordingly. The following pages provide a point-by-point answer to the comments raised by reviewers. All new corrections are written in blue theme in the revised manuscript. In this letter, the reviewer comments are indicated by the color blue, and the responses to comments are indicated by the color red.

Best regards,

For the authors,

Chenangnon Tovissode

Reviewer #1

This project cleverly uses evidence gathering behavior and reflection tests to find new evidence that insufficient evidence gathering can be explained by unreflective heuristics such as base rate neglect. I like how the authors draw on and return insight to multiple fields in cognitive and health sciences. Their data, materials, and paper seem to be worth publishing. However, I hope the editor will request some minor revisions (or else clarifications) about the following items:

Comment 1. "Reflection". This term is used many times in every section of the paper, but almost nothing about the construct, measure(s), or validation is mentioned until the Discussion section on page 11. I recommend defining the construct --e.g., "conscious and deliberate reconsideration of an initial intuition" (Byrd 2025) -- and the process tracing evidence showing that reflection tests/scores usually track such conscious and deliberate reasoning (Byrd et al. 2023).

Response

We thank the reviewer for pointing out the lack of such an important clarification. We have added the definition of *cognitive reflection* (line 137). We also describe how we measured this in the Methods (lines 280-299).

Comment 2. Table V reports Silhouette values for unsupervised clustering are GREATER THAN or EQUAL TO (.15, .16, .18, .18, .15) the values for supervised clustering (.11, .08, .09, .11, .15). Its caption indicates this means that unsupervised clustering yielded MORE well-separated observations (values closer to 1). However, the paper seems to report the opposite: "unsupervised clustering actually results in LESS well-separated... clusters". So there seems to be an error in the caption or interpretation of the cluster validation. Given that this reported result was used to argue for the use of the SHAP values in the analysis, this seems to require correction or clarification. If in fact the unsupervised clustering yielded superior separation, then it would seem (according to the logic of the paper), the analysis and results may need to be adjusted (according to the unsupervised clustering).

Response

Both the caption and the interpretation are correct. The relevant Silhouette values (comparing unsupervised and supervised clustering results) are in Table IV. There, we have the sequence (0.28 0.27 0.27 0.22 0.20) for unsupervised and (0.56 0.57 0.54 0.53 0.52) for supervised clustering: unsupervised clustering results in less well-separated clusters.

The Table V illustrates the fact that supervised clustering discards all information not useful for predicting Z and focuses on the main information during clustering. The table compares *unsupervised* hierarchical clustering on multiple correspondence analysis, using only the selected evidence data, versus using a combination of evidence data and the response Z. Both these approaches produce less well separated and less stable clusters, as compared to supervised clustering. In addition, directly including the response Z in unsupervised clustering results in less well separated and less stable clusters, as compared to not including Z. Hence the SHAP methodology is not simply adding information from the response Z, but relies on the main information in evidence that correlate with the response, discarding any residual information.

Comment 3. Another simple and potentially obvious test of something like Hypothesis 1 is that correct reflection test answers will predict significantly higher odds of choosing secondary evidence (compared to not) when given the opportunity t gather additional

evidence. I was surprised not to find this analysis or plot in the paper (or its supplement). Perhaps I missed it or there is a reason against this test that I have failed to consider.

Response
We thank the reviewer for this valuable comment. Looking at whether "correct reflection test answers will predict significantly higher odds of choosing secondary evidence (compared to not) when given the opportunity to gather additional evidence" was one of our objectives when designing the survey. Unfortunately, not many respondents took the opportunity to gather additional evidence: only 25% (683/2730) of respondents who could have selected more ev have taken this opportunity (now moved from the Appendix to the main text, see lines 314-318). In addition, this 25% is shared between the four target ev1-ev4, and other sources (ev5-ev12). Hence, any test based on these data will have limited power due to limited samples.

Looking at the "odds of choosing secondary evidence" is a quite different claim from H1 which is about the likelihood to seek all ev1-ev4. There is however a simpler test of H1: regressing the number of ev1-ev4 selected by respondents (or alternatively, whether they selected all four or not) on the reflection test score. We did not include this test results because it implies that we ignore other ev in the full landscape of evidence that we presented respondents with. We have now included this result in the Appendix, and refer to it in the main test, as an alternative to the used approach.

Comment 4. The paper states, "It is obviously not possible to directly measure which information respondents actually processed in such an information-diverse setting,....". I agree that THIS PAPER'S methods cannot directly measure what information was processed. However, process tracing researchers have a variety of methods for directly measuring what information people consider —- e.g., online think aloud protocols (Byrd et al. 2023), eye-tracking (Purcell et al. 2021), or even written reflection prompting (Cullen et al., 2022). Fortunately, this sentence is easily revised into a point about opportunities for further research: "Our design did not allow direct measurement of what information respondents considered, but process tracing methods could be used to triangulate on the unreflective base rate neglect we seem to have detected (Byrd et al....)."

Response
We thank the reviewer for pointing to these approaches to triangulate the finding. We have added the proposed modification into the limitations and future directions (see lines 622-626).

Typos? (see CAPS)
- "Fig 3.... evidence proposed to respondents MISSING PERIOD?..."
- "Fig 3.... groupS labels refer to...."
- "Fig 4.... respondents (N = 2905)BERT as a function of..."
- "gender... MALE" — 'man' seems more apt (unless the online survey item asked about 'sex'). This gender/sex mixup appears throughout the paper.

Response
We are grateful to the reviewer for catching these typos. We fixed them all.

Minor issues
A. The statistical analysis section exhibited lots of redundancy with the second half of the intro (and with the supplementary materials). It felt almost as if these sections were written independently and then combined without removing overlapping content. This is not catastrophic, but it may frustrate readers a bit.
B. I found the supplement to contain info I consider essential (Sections 1.1-1.3) and was surprised not to find it in the main text. If this was to satisfy an arbitrary word limit, then perhaps

the above-mentioned redundancy could be removed to make room for this seemingly important info in the main text.

Response
We have removed the redundant information from the Introduction. We also remove bring some of the material therein to the main text as suggested (see section Methods), further reducing the redundancy in the supplementary materials.

C. Plots.
- The labels were often just the strings of characters from the dataset (e.g., "india_health_authorities") rather than normally formatted text (e.g., "India health authorities"). This made the plots seem unfinished.
- The plots in the manuscript I received were pixelated. I hope the published version will feature the vector-graphic versions of the plots that R and Python can provide (e.g., PDF or SVG output). Vector graphics ensure infinite resolution and even encode the text into the image so that labels are machine readable, searchable, annotat-able, etc. So vector graphics are needed for accessibility (and not merely for, say, aesthetics).

Response
We used the suggested text format for the figure labels, and generated SVG outputs for the plots.

Reviewer #2

This paper presents a novel analytical approach, supervised clustering, to investigating patterns of evidence seeking behaviours, a timely and relevant topic. The authors refer to major literature in the field and present an interesting paradigm with a large amount of data. However, the structure of the argumentation and lack of support for the claims of the article make it unsuitable for publication at the current time.

Comment block 1
Abstract:
- The abstract lacks a typical structure of clearly and briefly presenting the main research question and findings. It introduces several terms without defining them.

Response
We have restructured the abstract to the main research question and findings. Note that we first identify a methodological challenge in evidence gathering and information processing and propose a solution. Then, as a case study, we identify a research question and apply our solution to demonstrate the fruitfulness of the approach. We also give the definition of each term. Below is the revised abstract.

Comment block 2

Introduction:

- Line 13: this is a good point about research needing to "zoom in" by controlling variables and how that affects ecological validity. It is also great to use a topic with "skin in the game."

- The emphasis on the idea of information processing and evidence gathering having "analogs" is unclear to me--why is that an important question, or why should it be phrased that way? This idea is not brought back up in the discussion.

Response
We now explicitly point to that in the discussion (lines 560-562) and added a reference to that in the introduction: <<In the *Discussion* section, we return to this and its importance for designing tailored communication so as to avoid fallacious inferences.>>

Comment block 3

- At several points it seems like the main research gap is about the lack of study on deference behavior, but you never define deference and it's not emphasized as a main question in the title, abstract, or literature review. If it is a main idea, it needs to be defined and reviewed. In other places, the emphasis is on heuristics, but they are likewise not fully introduced.

Response
The focus is on how people navigate the complex landscapes of evidence to make decisions, including the role of deference behavior, and use heuristics. We have added explanations of concept mentioned in the abstract and the introduction (including “deference” and “heuristics” among others).

Comment block 4

- "Expert source" is never defined in the context of your work

- Similarly, line 129 introduces the idea of cognitive reflection, a complex topic. It is never defined or reviewed and you do not explain the relationship between cognitive reflection and heuristic reasoning.

- In general, the theoretical background is very weak. All terms need definitions and important concepts need more space in the review, especially since these topics are very complex and their definitions have sometimes been unclear in prior literature.

- It would help to add subsections to the introduction to introduce the reader to the different ideas and organize your aims and predictions in a "current study" section.

Response

In general, we largely expanded the introduction to include definitions and review of important concepts. In particular, we have added the definition of “Expert source” and reviewed it, in relation to deference. (lines 58-71) and defined ‘‘cognitive reflection’’ (lines 137-138). We also added subsections to the introduction to organize the research gap and our contributions.

Comment block 5

Methods, Results:

- The data being a convenience sample collected online over a single day is of course a limitation. You mention that it is a representative sample, but in supplement section 1.1 it seems that this claim is based on the numbers just being similar to census data, without a formal analysis. There are only two gender categories and three political affiliation categories, which, although is probably based on the restrictions of the census data, is also a limitation.

Response

We removed “representative sample” from the Appendix where it appeared. We have also discussed the limitations of voluntary response sampling (lines 613-615). In regard to gender, our questionnaire offered the options: “Male”, “Female”, “Non-binary / third gender”, and “Prefer not to say”.

Comment block 6

- You did not find effects of political ideology, so this idea does not need to be emphasized, but you do need to briefly introduce why you might think there may be an effect (or a confounding effect) based on previous research.

Response

We added a paragraph on “political ideology” in the Methods section (lines 300-307). We explain why one might in general expect an effect based on previous research, but also how our specific design choices were made to minimize any confounding effect of political ideology.

Comment block 7

- Where did the items in supplement table II come from? Was there any piloting of the items?

Response
There was no piloting of the items. Items ev1-ev4 are simply the content of a 2 x 2 contingency table, with number chosen so that the use of heuristic processing is likely to result in a wrong conclusion. Items ev5-ev12 were selected to represent a diversity of evidence different from the 2 x 2 contingency table, appealing to some authoritative source (manufacturer, scientists, doctors, …). The items have been moved to the Methods in the main text (Table 2).

Comment block 8

-ev1-ev4 are very similar and at least conceptually complementary pairwise (we don't know how many total employees the company has, so maybe the math doesn't add up), whereas the rest of the items are more independent from each other. How does this affect the participant behavior and the analysis? You mention that some of those items are correlated (line 276) so it seems intentional. It is also unclear how the base rate neglect and confounders neglect heuristics apply to these 4 evidence items vs the rest of the items in terms of how respondents use the information, especially when they can control how many pieces of evidence they are exposed to.

Response
The total number of employees is the sum of the numbers in ev1-ev4, and we specified this at line 172 in the sentence: <<Respondents were informed that “a few months ago, a major midwestern company offered this new nasal spray for free to *all of its employees*. Last week, the firm conducted a survey of its employees. In this poll, people were asked if they used the nasal spray as directed and if they have since gotten sick with COVID-19.”>>
The pieces of evidence ev1-ev4 form together the full “statistical” type of evidence. They are indeed correlated. One of our objectives was to offer more fine-grained evidence and observe how people select from these pieces of evidence while keeping the possibility to select other pieces of evidence (deference). The base rate neglect and confounders neglect are precisely defined for these four evidence items. We now explain the composition of the evidence bank in the Methods (lines 260-267).

Comment block 9

- CRT is in Table 1 and in the analysis but is not mentioned in the methods or discussed in the introduction, which is necessary as again it is a complex topic with some debate over what the CRT actually indexes.

Response
We have added a description of the CRT in the Methods, with details on how it is measured (lines 289-299).

Comment block 10

- The idea of "first-order evidence" and how it relates to "deference" is unclear throughout the article; e.g. line 286-288 it is unclear what is meant by "conclusive evidence by deference." If there are some evidence items that are categorized as belonging to "deference," then this needs to be explained.

Response
We have defined the terms first-order evidence" and "deference" and discussed their relation in the introduction (lines 58--71). The phrase "conclusive evidence by deference" refers to any piece of evidence from ev5-ev12, that points to the effectiveness of the nasal spray (e.g. ev12). We now explain the composition of the evidence bank in the Methods (lines 260-267).

Comment block 11

- Generally it is unclear whether you intend to use political ideology as a main predictor or a confounder. If a main predictor, it should be given more discussion.

Response
Political ideology is considered as a potential confounder, based on literature review, but expected to not affect our outcome, because of the design choice we made on purpose for that.

Comment block 12

- Supplement figure II refers to "primary" and "secondary" evidence, but these terms are not used or discussed anywhere in the main article although the idea of secondary evidence is mentioned once in the introduction.

Response
We have defined the terms "primary" and "secondary" evidence in the Methods (lines 270-279), and explain why our analyses did not focus on this distinction (lines 313-318).

Comment block 13

Discussion:

- Throughout the paper, “folks” is used interchangeably with “respondents”—"folks” is nonstandard for academic English and may imply people outside the participants of the study.

Response
We removed the term “folk” from the Appendix where it appeared.

Comment block 14

- Line 359 says that the focus of the paper is “how people navigate data that need processing versus deference to outside sources” but this is not clear in the actual paper.

Response
We have clarified in the Abstract and the Introduction that our focus is on how people navigate the complex landscapes of evidence, e.g. how people might combine different sources such as first order data and deference to experts deemed relevant. We have clearly identified our main research gap from the study of this navigation (Lines 12-24).

Comment block 15

- Lines 366-388: for me, the claims of ecological validity and granularity are too strong. There are a limited set of items here, in what is essentially a restricted exploration space and a fictional scenario. Other studies have clearly controlled for deference to different types of authorities, trustworthiness, scientific validity of evidence, etc. and the results of this work are not contextualized with those recent studies.

Response

We are particularly grateful to the reviewer for this comment. We have tempered the claims on ecological validity and granularity in the Discussion section and recognize the limitations of our study in respect to the explored items and the fictious scenario.

Comment block 16

- Line 447+: Is this discussion about categorical and associative standards of evidence proposing it as a new typology? Or do these terms come from somewhere else? Are they intended to be the converse of each other (they are not)? Is the associative standard not similar to existing theories of evidence evaluation using multiple sources?

Response

We have now introduced the terms *categorical* and *associative* standards in the introduction (lines 39-57), as part of the description of the current state of evidence typologies. In particular, these terms have been discussed in the context of information seeking behaviors is a recent study (Justwan et al., 2025). In the Discussion, we are not opposing categorical and associative standards. We are suggesting that studies consider typologies of evidence that include more fine-grained first-order data to allow the distinction between categorical and associative standards, as opposed to the current broad “statistical” evidence type. Our results suggest that allowing these distinctions can provide a hint at how people use first order data, irrespective of whether they combine it with information from other sources.
Theories of evidence evaluation using multiple sources also study how people navigate the complex landscapes of evidence, for instance, in presence of sources with varying level of expertise, trustworthiness, authority. However, while we maintain people in a landscape of evidence from multiple sources, we are especially interested in what first-order data they seek, what they do with it, to approach how they use it.

Justwan, F., & Baumgaertner, B. (2025). The effects of ideology and cognitive reflection on evidence gathering behavior in the political domain. *Plos one*, 20(12).

Comment block 17

- Throughout the paper, I am missing some details, definitions and connections between the ideas of evidence seeking/gathering, information processing, deference, heuristic reasoning, and cognitive reflection. In general, the claims are not adequately supported.

Response

We have included the definitions and connections between the ideas of evidence seeking/gathering, information processing, deference, heuristic reasoning, and cognitive reflection throughout the Introduction. We have also tempered our claims about the ecological

validity by explicitly recognizing the limitations of our sample and study design, and our choice of application domain (lines 614-631).

Comment block 17

Limitations:

- a major limitation is the simplicity of your items

Response

We recognized the simplicity of our items given the complexity of a real landscape of evidence people face in practice (lines 615-625). We point to studies where more extensive information landscapes were considered as the next target to apply our proposed methodology.

Comment block 18

- many of the “future directions” you suggest have already been done, such as allowing participants to do free web searches or more freely explore realistic and more extensive information platforms, and including misinformation and conflicting information. In light of that, how is this work justified as a novel contribution to the field? I would focus more on the real novelty—the analytical approach.

Response

Our contribution to the field is indeed the analytical approach. We have restructured the introduction to clarify this. We suggest to explore “future directions”, using our proposed methodology. These studies already have the potential of fine-grained evidence typology and would benefit from an application of our methodology.

Comment block 19

In general, this paper presents a potentially useful analytical method, but the ideas, reasoning flow, and contextualization within the literature of the field need much more work.

Response

We thank the reviewer for this comment. We have introduced more literature to contextualize the study, define concepts, and relate them. We clearly identified the analytical gap as our main target, both in the abstract and at the beginning of the introduction. We also provide a more detailed description of the study design and methodology in the main text.
