# Supplementary material for: Patterns between evidence seeking behaviors, reasoning, and cognitive reflection: A supervised clustering approach
Source: PLoS One. 2026 Jun 25;21(6):e0352096. doi: 10.1371/journal.pone.0352096 (PMC13298786; doi:10.1371/journal.pone.0352096)
Supplement: Attachment — Submitted filename: Response_to_Reviewers.pdf [file pone.0352096.s008.pdf]

Dear Editor of Plos One,

I am submitting this letter in response to the decision: Revision required [PONE-D-25-61437R1] (by July 03, 2026).

Thank you for considering our revisions and giving us the opportunity to improve our manuscript. We are grateful to the two reviewers for their comments and constructive suggestions to improve the abstract and introduction. We provide a point-by-point answer to reviewers' comments in the following pages. The reviewers' comments are indicated by the color blue, and the responses to comments are indicated by the color red.

Best regards,

For the authors,

Chenangnon Tovissode

Reviewer #1

I found the revisions satisfactory and, thus, can recommend them for publication. I also appreciated some of the improvements that Reviewer 2 elicited.

Comment. If, however, the editor were to request further revision, I think one very minor opportunity to improve the paper would involve explicit integration of the reflection test result(s) into the paper's abstract. As far as I can tell, the fact that reflection test performance predicts less neglect of relevant evidence provides some independent evidence of some of the authors' claims. So without explicit mention of what reflection test performance predicts, the abstract may not relay as much of the paper's supporting evidence as it could.

Response

We thank the reviewer for pointing out the lack of this important result in the abstract. We have explicitly included the reflection test results as independent evidence supporting our conclusion, by adding the following phrase: <<... we find that higher performance on the cognitive reflection test predicts selection of the full data of a 2 x 2 contingency table.>>

Reviewer #2

I thank the authors for addressing my comments and commend them for improving their paper immensely. I have only a minor revision request at this point.

The abstract is now much more clear and the goals of the paper are clearer as well.

Comment block 1

However, the concept of *"deference"* still comes into the abstract at the very end, with no mention of what it is or why it is involved with the main results. Additionally, the sentence *"Consequently, evidential categories in information seeking research are likely overly monolithic, despite their advantage of studying a wider range of evidential categories used in decision making"* is difficult to understand.

Response

We have revised the abstract to include these two sentences which provide an inline definition of deference and clarify the unclear sentence: <<*But, the latter group is nevertheless similar to base rate neglecters in who they consider to be relevant outside sources (i.e., they have roughly the same ``deference'' behavior). So while evidential categories in information seeking research are well suited to track differences in deference behaviors, they are blind to the difference between these two groups.*>> This also clarifies why *"deference"* is involved in the main results.

Comment block 2

The introduction is much improved! The new structure greatly helps clarify your position and goals and addressed most of my comments.

I would still need more of an actual definition for "deference"--line 70; if it is an important construct in the paper, it should be defined explicitly, not obliquely. I appreciate that the distinctions between "first-order" and "second-order" evidence are mixed in the literature. But it is also still a bit unclear to me how "deference" differs from theories of epistemic justifications as you've mentioned briefly, e.g. justification by authority (for example, Meng-Jung Tsai, Ching-Yeh Wang, An-Hsuan Wu, Ivar Bråten, Differences in epistemic justification profiles during conflicting socio-scientific information processing: A study of visual and memory-based behavior via eye-tracking, Acta Psychologica, Volume 252, 2025, https://doi.org/10.1016/j.actpsy.2024.104680. (https://www.sciencedirect.com/science/article/pii/S0001691824005584). And additionally it is unclear where the construct comes from. Is it a novel idea meant as an umbrella for several related constructs? Or something coming from previous literature? You mention that you use deference to avoid making assumptions about who participants would believe are "experts" (and thus, authorities?).

The ideas are almost there in the discussion around lines 58-79, but it is still unclear how deference relates to the existing concept of a "source feature" of evidence, which as I understand it, would be related to second-order evidence. It is very important to be clear about your claims about this construct, since your analytical approach uses weights on information *sources*. Deference would then be directly related to the "source features" as participants understand them, e.g. authority, trustworthiness, expertise, etc.

Overall, this manuscript has been greatly improved. I only have the last comments about the importance of the "deference" construct in relation to the authors' claims and use of it and its relationship to existing similar constructs. If the authors would address this issue, then I would find the paper suitable to publish.

Response
We greatly appreciate this comment which prompted us to clarify the term "deference" in the paper. The concept of *deference* in this work is not a construct; hence we do not make a comparison with existing theories of epistemic justifications. It is not a novel idea meant as an umbrella for several related constructs. By deference, we simply mean *consideration of pre-processed information as relevant*. Here is the complete revised paragraph where we introduce the term in the introduction:

<< What we would like to do is offer people, in a controlled setting, a wider and more fine-grained selection of evidence that spans both gathering and processing research. For the processing side, we will use “statistical” to mean the entries in a contingency table (but unprocessed, so to speak). This enables us to detect the possibilities of processing heuristics. On the information gathering side we will use “deference” to mean sources where information has been pre-processed or given an interpretation (e.g. a recommendation) and seen as potentially relevant without the presumption that subjects see the sources as experts. Now if we give subjects even just 12 options to select from across these two sides, we face the problem of combinatorial explosion that the typologies of evidence are meant to make tractable, especially if we consider the full pathway of gathering information and its processing (e.g. by making a judgment about causal efficacy). In the next section we illustrate an analytical approach for handling this challenge. >>

This is indeed related to *second-order evidence*, and to many concepts in the literature, as we discussed when comparing our empirical results to existing theoretical delineations of evidence categories.
