# Supplementary material for: Patterns between evidence seeking behaviors, reasoning, and cognitive reflection: A supervised clustering approach
Source: PLoS One. 2026 Jun 25;21(6):e0352096. doi: 10.1371/journal.pone.0352096 (PMC13298786; doi:10.1371/journal.pone.0352096)
Supplement: Peer review history

# Peer review: Decision Letter 0

**DOI:** 10.1371/journal.pone.0352096.r001

**Authors:** Fernando Blanco

10 Mar 2026

PONE-D-25-61437

Patterns between evidence seeking behaviors, reasoning, and cognitive reflection: a supervised clustering approach

PLOS One

Dear Dr. Tovissodé,

Thank you for submitting your manuscript to PLOS ONE. After careful consideration, we feel that it has merit but does not fully meet PLOS ONE’s publication criteria as it currently stands. Therefore, we invite you to submit a revised version of the manuscript that addresses the points raised during the review process.

Please submit your revised manuscript by Apr 24 2026 11:59PM. If you will need more time than this to complete your revisions, please reply to this message or contact the journal office at plosone@plos.org. When you're ready to submit your revision, log on to https://www.editorialmanager.com/pone/ and select the 'Submissions Needing Revision' folder to locate your manuscript file.

Please include the following items when submitting your revised manuscript:

- A letter that responds to each point raised by the academic editor and reviewer(s). You should upload this letter as a separate file labeled 'Response to Reviewers'.
- A marked-up copy of your manuscript that highlights changes made to the original version. You should upload this as a separate file labeled 'Revised Manuscript with Track Changes'.
- An unmarked version of your revised paper without tracked changes. You should upload this as a separate file labeled 'Manuscript'.

If you would like to make changes to your financial disclosure, please include your updated statement in your cover letter. Guidelines for resubmitting your figure files are available below the reviewer comments at the end of this letter.

If applicable, we recommend that you deposit your laboratory protocols in protocols.io to enhance the reproducibility of your results. Protocols.io assigns your protocol its own identifier (DOI) so that it can be cited independently in the future. For instructions see: https://journals.plos.org/plosone/s/submission-guidelines#loc-laboratory-protocols. Additionally, PLOS ONE offers an option for publishing peer-reviewed Lab Protocol articles, which describe protocols hosted on protocols.io. Read more information on sharing protocols at https://plos.org/protocols?utm_medium=editorial-email&utm_source=authorletters&utm_campaign=protocols.

We look forward to receiving your revised manuscript.

Kind regards,

Fernando Blanco

Academic Editor

PLOS One

Journal Requirements:

When submitting your revision, we need you to address these additional requirements.

1.Please ensure that your manuscript meets PLOS ONE's style requirements, including those for file naming. The PLOS ONE style templates can be found at

https://journals.plos.org/plosone/s/file?id=wjVg/PLOSOne_formatting_sample_main_body.pdf and

https://journals.plos.org/plosone/s/file?id=ba62/PLOSOne_formatting_sample_title_authors_affiliations.pdf

2. Please note that PLOS One has specific guidelines on code sharing for submissions in which author-generated code underpins the findings in the manuscript. In these cases, we expect all author-generated code to be made available without restrictions upon publication of the work. Please review our guidelines at https://journals.plos.org/plosone/s/materials-and-software-sharing#loc-sharing-code and ensure that your code is shared in a way that follows best practice and facilitates reproducibility and reuse.

3.  Thank you for stating the following financial disclosure:

“Research by C.F.T., F.J. and B.B. reported in this publication was supported by the National Institute Of General Medical Sciences of the National Institutes of Health (https://www.nigms.nih.gov) under Award Number P20GM104420. The content is solely the responsibility of the authors and does not necessarily represent the official views of the National Institutes of Health.”

Please state what role the funders took in the study.  If the funders had no role, please state: "The funders had no role in study design, data collection and analysis, decision to publish, or preparation of the manuscript."

If this statement is not correct you must amend it as needed.

Please include this amended Role of Funder statement in your cover letter; we will change the online submission form on your behalf.

4. If the reviewer comments include a recommendation to cite specific previously published works, please review and evaluate these publications to determine whether they are relevant and should be cited. There is no requirement to cite these works unless the editor has indicated otherwise.

Additional Editor Comments:

First of all, I would like to apologize for the delay in making a decision. It has been particularly difficult to obtain two reviews from experts in the field, and this problem is becoming more common in recent times.

Finally, I have received the two expert reports and I believe that, although they recognize the merit and interest of some aspects of the work (especially the analytical strategy) there are areas for improvement that should be addressed in order to allow the publication of the article.

First, the Introduction section should explain all the relevant concepts needed to understand the research presented (e.g., reflection…) including the appropriate references. Both reviewers agree on this point.

Similarly, in other sections of the article there is a lack of detail necessary to fully understand what is being presented (e.g., sampling procedures). A rewriting is required in order to incorporate this information.

It is also necessary to incorporate some comments (mainly those specifically raised by Reviewer 2) as limitations of the study: for example, how the present proposal fits within a body of literature in which more ecological tests of online information search already exist. The Discussion section needs to be overhauled to include a deeper discussion of the results and the limitations.

In general, I believe that most or all of the reviewrs’ comments can be incorporated into the article without the need to obtain new data, by rewriting it to: (a) include all the necessary information and references to understand and contextualize the research, (b) clearly present the issues of interpretation and generalization described by the reviewers as limitations (Discussion section). Both reviewers have provided useful ideas to achieve this.

[Note: HTML markup is below. Please do not edit.]

Reviewer's Responses to Questions

**Comments to the Author**

1. Is the manuscript technically sound, and do the data support the conclusions?

The manuscript must describe a technically sound piece of scientific research with data that supports the conclusions. Experiments must have been conducted rigorously, with appropriate controls, replication, and sample sizes. The conclusions must be drawn appropriately based on the data presented.

Reviewer #1: Partly

Reviewer #2: Partly

**********

2. Has the statistical analysis been performed appropriately and rigorously?

Reviewer #1: I Don't Know

Reviewer #2: Yes

**********

3. Have the authors made all data underlying the findings in their manuscript fully available?

The PLOS Data policy requires authors to make all data underlying the findings described in their manuscript fully available without restriction, with rare exception (please refer to the Data Availability Statement in the manuscript PDF file). The data should be provided as part of the manuscript or its supporting information, or deposited to a public repository. For example, in addition to summary statistics, the data points behind means, medians and variance measures should be available. If there are restrictions on publicly sharing data—e.g. participant privacy or use of data from a third party—those must be specified.

Reviewer #1: Yes

Reviewer #2: Yes

**********

4. Is the manuscript presented in an intelligible fashion and written in standard English?

PLOS ONE does not copyedit accepted manuscripts, so the language in submitted articles must be clear, correct, and unambiguous. Any typographical or grammatical errors should be corrected at revision, so please note any specific errors here.

Reviewer #1: Yes

Reviewer #2: Yes

**********

5. Review Comments to the Author

Please use the space provided to explain your answers to the questions above. You may also include additional comments for the author, including concerns about dual publication, research ethics, or publication ethics. (Please upload your review as an attachment if it exceeds 20,000 characters)

Reviewer #1: This project cleverly uses evidence gathering behavior and reflection tests to find new evidence that insufficient evidence gathering can be explained by unreflective heuristics such as base rate neglect. I like how the authors draw on and return insight to multiple fields in cognitive and health sciences. Their data, materials, and paper seem to be worth publishing. However, I hope the editor will request some minor revisions (or else clarifications) about the following items:

1. "Reflection". This term is used many times in every section of the paper, but almost nothing about the construct, measure(s), or validation is mentioned until the Discussion section on page 11. I recommend defining the construct -- e.g., "conscious and deliberate reconsideration of an initial intuition" (Byrd 2025) —- and the process tracing evidence showing that reflection tests/scores usually track such conscious and deliberate reasoning (Byrd et al. 2023).

2. Table V reports Silhouette values for unsupervised clustering are GREATER THAN or EQUAL TO (.15, .16, .18, .18, .15) the values for supervised clustering (.11, .08, .09, .11, .15). Its caption indicates this means that unsupervised clustering yielded MORE well-separated observations (values closer to 1). However, the paper seems to report the opposite: "unsupervised clustering actually results in LESS well-separated... clusters". So there seems to be an error in the caption or interpretation of the cluster validation. Given that this reported result was used to argue for the use of the SHAP values in the analysis, this seems to require correction or clarification. If in fact the unsupervised clustering yielded superior separation, then it would seem (according to the logic of the paper), the analysis and results may need to be adjusted (according to the unsupervised clustering).

3. Another simple and potentially obvious test of something like Hypothesis 1 is that correct reflection test answers will predict significantly higher odds of choosing secondary evidence (compared to not) when given the opportunity to gather additional evidence. I was surprised not to find this analysis or plot in the paper (or its supplement). Perhaps I missed it or there is a reason against this test that I have failed to consider.

4. The paper states, “It is obviously not possible to directly measure which information respondents actually processed in such an information-diverse setting,....". I agree that THIS PAPER'S methods cannot directly measure what information was processed. However, process tracing researchers have a variety of methods for directly measuring what information people consider —- e.g., online think aloud protocols (Byrd et al. 2023), eye-tracking (Purcell et al. 2021), or even written reflection prompting (Cullen et al., 2022). Fortunately, this sentence is easily revised into a point about opportunities for further research: "Our design did not allow direct measurement of what information respondents considered, but process tracing methods could be used to triangulate on the unreflective base rate neglect we seem to have detected (Byrd et al....)."

Typos? (see CAPS)

- "Fig 3.... evidence proposed to respondents MISSING PERIOD?..."

- "Fig 3.... groupS labels refer to...."

- "Fig 4.... respondents (N = 2905)BERT as a function of..."

- "gender... MALE" — 'man' seems more apt (unless the online survey item asked about 'sex'). This gender/sex mixup appears throughout the paper.

Minor issues

A. The statistical analysis section exhibited lots of redundancy with the second half of the intro (and with the supplementary materials). It felt almost as if these sections were written independently and then combined without removing overlapping content. This is not catastrophic, but it may frustrate readers a bit.

B. I found the supplement to contain info I consider essential (Sections 1.1-1.3) and was surprised not to find it in the main text. If this was to satisfy an arbitrary word limit, then perhaps the above-mentioned redundancy could be removed to make room for this seemingly important info in the main text.

C. Plots.

- The labels were often just the strings of characters from the dataset (e.g., "india_health_authorities") rather than normally formatted text (e.g., "India health authorities"). This made the plots seem unfinished.

- The plots in the manuscript I received were pixelated. I hope the published version will feature the vector-graphic versions of the plots that R and Python can provide (e.g., PDF or SVG output). Vector graphics ensure infinite resolution and even encode the text into the image so that labels are machine readable, searchable, annotat-able, etc. So vector graphics are needed for accessibility (and not merely for, say, aesthetics).

Nick Byrd

(I sign all of my reviews)

References

Byrd, N. (2025). A Two-Factor Explication of “Reflection”: Unifying, Making Sense of, and Guiding the Philosophy and Science of Reflective Reasoning. Res Philosophica, 102(3), 373–392. https://doi.org/10.5840/resphilosophica2711

Byrd, N., Joseph, B., Gongora, G., & Sirota, M. (2023). Tell Us What You Really Think: A Think Aloud Protocol Analysis of the Verbal Cognitive Reflection Test. Journal of Intelligence, 11(4), Article 4. https://doi.org/10.3390/jintelligence11040076

Cullen, S., Byrd, N., Chapkovski, P., & Thomason, N. (2022). Thinking Alone, and Together: Dissenting Pairs Corrected More Faulty Decisions Than Solitary Reasoners Across Four Tasks. Reflection on intelligent systems: towards a cross-disciplinary definition, Universität Stuttgart. https://www.researchgate.net/publication/390335508

Purcell, Z. A., Howarth, S., Wastell, C. A., Roberts, A. J., & Sweller, N. (2021). Eye tracking and the cognitive reflection test: Evidence for intuitive correct responding and uncertain heuristic responding. Memory & Cognition. https://doi.org/10.3758/s13421-021-01224-8

Reviewer #2: This paper presents a novel analytical approach, supervised clustering, to investigating patterns of evidence seeking behaviours, a timely and relevant topic. The authors refer to major literature in the field and present an interesting paradigm with a large amount of data. However, the structure of the argumentation and lack of support for the claims of the article make it unsuitable for publication at the current time.

Abstract:

- The abstract lacks a typical structure of clearly and briefly presenting the main research question and findings. It introduces several terms without defining them.

Introduction:

- Line 13: this is a good point about research needing to "zoom in" by controlling variables and how that affects ecological validity. It is also great to use a topic with "skin in the game."

- The emphasis on the idea of information processing and evidence gathering having "analogs" is unclear to me--why is that an important question, or why should it be phrased that way? This idea is not brought back up in the discussion.

- At several points it seems like the main research gap is about the lack of study on deference behavior, but you never define deference and it's not emphasized as a main question in the title, abstract, or literature review. If it is a main idea, it needs to be defined and reviewed. In other places, the emphasis is on heuristics, but they are likewise not fully introduced.

- "Expert source" is never defined in the context of your work

- Similarly, line 129 introduces the idea of cognitive reflection, a complex topic. It is never defined or reviewed and you do not explain the relationship between cognitive reflection and heuristic reasoning.

- In general, the theoretical background is very weak. All terms need definitions and important concepts need more space in the review, especially since these topics are very complex and their definitions have sometimes been unclear in prior literature.

- It would help to add subsections to the introduction to introduce the reader to the different ideas and organize your aims and predictions in a "current study" section.

Methods, Results:

- The data being a convenience sample collected online over a single day is of course a limitation. You mention that it is a representative sample, but in supplement section 1.1 it seems that this claim is based on the numbers just being similar to census data, without a formal analysis. There are only two gender categories and three political affiliation categories, which, although is probably based on the restrictions of the census data, is also a limitation.

- You did not find effects of political ideology, so this idea does not need to be emphasized, but you do need to briefly introduce why you might think there may be an effect (or a confounding effect) based on previous research.

- Where did the items in supplement table II come from? Was there any piloting of the items?

-ev1-ev4 are very similar and at least conceptually complementary pairwise (we don't know how many total employees the company has, so maybe the math doesn't add up), whereas the rest of the items are more independent from each other. How does this affect the participant behavior and the analysis? You mention that some of those items are correlated (line 276) so it seems intentional. It is also unclear how the base rate neglect and confounders neglect heuristics apply to these 4 evidence items vs the rest of the items in terms of how respondents use the information, especially when they can control how many pieces of evidence they are exposed to.

- CRT is in Table 1 and in the analysis but is not mentioned in the methods or discussed in the introduction, which is necessary as again it is a complex topic with some debate over what the CRT actually indexes.

- The idea of "first-order evidence" and how it relates to "deference" is unclear throughout the article; e.g. line 286-288 it is unclear what is meant by "conclusive evidence by deference." If there are some evidence items that are categorized as belonging to "deference," then this needs to be explained.

- Generally it is unclear whether you intend to use political ideology as a main predictor or a confounder. If a main predictor, it should be given more discussion.

- Supplement figure II refers to "primary" and "secondary" evidence, but these terms are not used or discussed anywhere in the main article although the idea of secondary evidence is mentioned once in the introduction.

Discussion:

- Throughout the paper, "folks" is used interchangeably with "respondents"--"folks" is nonstandard for academic English and may imply people outside the participants of the study.

- Line 359 says that the focus of the paper is "how people navigate data that need processing versus

deference to outside sources" but this is not clear in the actual paper.

- Lines 366-388: for me, the claims of ecological validity and granularity are too strong. There are a limited set of items here, in what is essentially a restricted exploration space and a fictional scenario. Other studies have clearly controlled for deference to different types of authorities, trustworthiness, scientific validity of evidence, etc. and the results of this work are not contextualized with those recent studies

- Line 447+: Is this discussion about categorical and associative standards of evidence proposing it as a new typology? Or do these terms come from somewhere else? Are they intended to be the converse of each other (they are not)? Is the associative standard not similar to existing theories of evidence evaluation using multiple sources?

- Throughout the paper, I am missing some details, definitions and connections between the ideas of evidence seeking/gathering, information processing, deference, heuristic reasoning, and cognitive reflection. In general, the claims are not adequately supported.

Limitations:

- a major limitation is the simplicity of your items

- many of the "future directions" you suggest have already been done, such as allowing participants to do free web searches or more freely explore realistic and more extensive information platforms, and including misinformation and conflicting information. In light of that, how is this work justified as a novel contribution to the field? I would focus more on the real novelty--the analytical approach.

In general, this paper presents a potentially useful analytical method, but the ideas, reasoning flow, and contextualization within the literature of the field need much more work.

**********

6. PLOS authors have the option to publish the peer review history of their article (what does this mean?). If published, this will include your full peer review and any attached files.

If you choose “no”, your identity will remain anonymous but your review may still be made public.

**Do you want your identity to be public for this peer review?**  For information about this choice, including consent withdrawal, please see our Privacy Policy.

Reviewer #1: **Yes:** Nick Byrd

Reviewer #2: No

**********

[NOTE: If reviewer comments were submitted as an attachment file, they will be attached to this email and accessible via the submission site. Please log into your account, locate the manuscript record, and check for the action link "View Attachments". If this link does not appear, there are no attachment files.]

To ensure your figures meet our technical requirements, please review our figure guidelines: https://journals.plos.org/plosone/s/figures

You may also use PLOS’s free figure tool, NAAS, to help you prepare publication quality figures: https://journals.plos.org/plosone/s/figures#loc-tools-for-figure-preparation.

NAAS will assess whether your figures meet our technical requirements by comparing each figure against our figure specifications.

# Author comment: Author response to Decision Letter 1

**DOI:** 10.1371/journal.pone.0352096.r002

23 Apr 2026

Dear Editor of Plos One,

I am submitting this letter in response to the decision: Revision required [PONE-D-25-61437] (by April 24, 2026).

Thank you for considering our submission and giving us the opportunity to revise our manuscript. As you required, we have added the following statement to the financial disclosure in the manuscript: “The funders had no role in study design, data collection and analysis, decision to publish, or preparation of the manuscript.” Moreover, after reviewing the publications suggested by the reviewers, we have cited those relevant to the revisions we made. We reorganized the introduction and included all the necessary information and references to understand and contextualize the research.

My co-authors and I are grateful to the two reviewers for the comments and constructive suggestions to improve both the content and the presentation of the manuscript. We thoroughly revised the manuscript accordingly. The following pages provide a point-by-point answer to the comments raised by reviewers. All new corrections are written in blue theme in the revised manuscript.

Best regards,

For the authors,

Chenangnon Tovissode

Reviewer #1

This project cleverly uses evidence gathering behavior and reflection tests to find new evidence that insufficient evidence gathering can be explained by unreflective heuristics such as base rate neglect. I like how the authors draw on and return insight to multiple fields in cognitive and health sciences. Their data, materials, and paper seem to be worth publishing. However, I hope the editor will request some minor revisions (or else clarifications) about the following items:

Comment 1. "Reflection". This term is used many times in every section of the paper, but almost nothing about the construct, measure(s), or validation is mentioned until the Discussion section on page 11. I recommend defining the construct --e.g., "conscious and deliberate reconsideration of an initial intuition" (Byrd 2025) -- and the process tracing evidence showing that reflection tests/scores usually track such conscious and deliberate reasoning (Byrd et al. 2023).

Response

We thank the reviewer for pointing out the lack of such an important clarification. We have added the definition of cognitive reflection (line 137). We also describe how we measured this in the Methods (lines 280-299).

Comment 2. Table V reports Silhouette values for unsupervised clustering are GREATER THAN or EQUAL TO (.15, .16, .18, .18, .15) the values for supervised clustering (.11, .08, .09, .11, .15). Its caption indicates this means that unsupervised clustering yielded MORE well-separated observations (values closer to 1). However, the paper seems to report the opposite: "unsupervised clustering actually results in LESS well-separated... clusters". So there seems to be an error in the caption or interpretation of the cluster validation. Given that this reported result was used to argue for the use of the SHAP values in the analysis, this seems to require correction or clarification. If in fact the unsupervised clustering yielded superior separation, then it would seem (according to the logic of the paper), the analysis and results may need to be adjusted (according to the unsupervised clustering).

Response

Both the caption and the interpretation are correct. The relevant Silhouette values (comparing unsupervised and supervised clustering results) are in Table IV. There, we have the sequence (0.28 0.27 0.27 0.22 0.20) for unsupervised and (0.56 0.57 0.54 0.53 0.52) for supervised clustering: unsupervised clustering results in less well-separated clusters.

The Table V illustrates the fact that supervised clustering discards all information not useful for predicting Z and focuses on the main information during clustering. The table compares unsupervised hierarchical clustering on multiple correspondence analysis, using only the selected evidence data, versus using a combination of evidence data and the response Z. Both these approaches produce less well separated and less stable clusters, as compared to supervised clustering. In addition, directly including the response Z in unsupervised clustering results in less well separated and less stable clusters, as compared to not including Z. Hence the SHAP methodology is not simply adding information from the response Z, but relies on the main information in evidence that correlate with the response, discarding any residual information.

Comment 3. Another simple and potentially obvious test of something like Hypothesis 1 is that correct reflection test answers will predict significantly higher odds of choosing secondary evidence (compared to not) when given the opportunity t gather additional evidence. I was surprised not to find this analysis or plot in the paper (or its supplement). Perhaps I missed it or there is a reason against this test that I have failed to consider.

Response

We thank the reviewer for this valuable comment. Looking at whether “correct reflection test answers will predict significantly higher odds of choosing secondary evidence (compared to not) when given the opportunity to gather additional evidence” was one of our objectives when designing the survey. Unfortunately, not many respondents took the opportunity to gather additional evidence: only 25% (683/2730) of respondents who could have selected more ev have taken this opportunity (now moved from the Appendix to the main text, see lines 314-318). In addition, this 25% is shared between the four target ev1-ev4, and other sources (ev5-ev12). Hence, any test based on these data will have limited power due to limited samples.

Looking at the “odds of choosing secondary evidence” is a quite different claim from H1 which is about the likelihood to seek all ev1-ev4. There is however a simpler test of H1: regressing the number of ev1-ev4 selected by respondents (or alternatively, whether they selected all four or not) on the reflection test score. We did not include this test results because it implies that we ignore other ev in the full landscape of evidence that we presented respondents with. We have now included this result in the Appendix, and refer to it in the main test, as an alternative to the used approach.

Comment 4. The paper states, “It is obviously not possible to directly measure which information respondents actually processed in such an information-diverse setting,....". I agree that THIS PAPER'S methods cannot directly measure what information was processed. However, process tracing researchers have a variety of methods for directly measuring what information people consider —- e.g., online think aloud protocols (Byrd et al. 2023), eye-tracking (Purcell et al. 2021), or even written reflection prompting (Cullen et al., 2022). Fortunately, this sentence is easily revised into a point about opportunities for further research: "Our design did not allow direct measurement of what information respondents considered, but process tracing methods could be used to triangulate on the unreflective base rate neglect we seem to have detected (Byrd et al....)."

Response

We thank the reviewer for pointing to these approaches to triangulate the finding. We have added the proposed modification into the limitations and future directions (see lines 622-626).

Typos? (see CAPS)

- "Fig 3.... evidence proposed to respondents MISSING PERIOD?..."

- "Fig 3.... groupS labels refer to...."

- "Fig 4.... respondents (N = 2905)BERT as a function of..."

- "gender... MALE" — 'man' seems more apt (unless the online survey item asked about 'sex'). This gender/sex mixup appears throughout the paper.

Response

We are grateful to the reviewer for catching these typos. We fixed them all.

Minor issues

A. The statistical analysis section exhibited lots of redundancy with the second half of the intro (and with the supplementary materials). It felt almost as if these sections were written independently and then combined without removing overlapping content. This is not catastrophic, but it may frustrate readers a bit.

B. I found the supplement to contain info I consider essential (Sections 1.1-1.3) and was surprised not to find it in the main text. If this was to satisfy an arbitrary word limit, then perhaps the above-mentioned redundancy could be removed to make room for this seemingly important info in the main text.

Response

We have removed the redundant information from the Introduction. We also remove bring some of the material therein to the main text as suggested (see section Methods), further reducing the redundancy in the supplementary materials.

C. Plots.

- The labels were often just the strings of characters from the dataset (e.g., "india_health_authorities") rather than normally formatted text (e.g., "India health authorities"). This made the plots seem unfinished.

- The plots in the manuscript I received were pixelated. I hope the published version will feature the vector-graphic versions of the plots that R and Python can provide (e.g., PDF or SVG output). Vector graphics ensure infinite resolution and even encode the text into the image so that labels are machine readable, searchable, annotat-able, etc. So vector graphics are needed for accessibility (and not merely for, say, aesthetics).

Response

We used the suggested text format for the figure labels, and generated SVG outputs for the plots.

Reviewer #2

This paper presents a novel analytical approach, supervised clustering, to investigating patterns of evidence seeking behaviours, a timely and relevant topic. The authors refer to major literature in the field and present an interesting paradigm with a large amount of data. However, the structure of the argumentation and lack of support for the claims of the article make it unsuitable for publication at the current time.

Comment block 1

Abstract:

- The abstract lacks a typical structure of clearly and briefly presenting the main research question and findings. It introduces several terms without defining them.

Response

We have restructured the abstract to the main research question and findings. Note that we first identify a methodological challenge in evidence gathering and information processing and propose a solution. Then, as a case study, we identify a research question and apply our solution to demonstrate the fruitfulness of the approach. We also give the definition of each term. Below is the revised abstract.

Comment block 2

Introduction:

- Line 13: this is a good point about research needing to "zoom in" by controlling variables and how that affects ecological validity. It is also great to use a topic with "skin in the game."

- The emphasis on the idea of information processing and evidence gathering having "analogs" is unclear to me--why is that an important question, or why should it be phrased that way? This idea is not brought back up in the discussion.

Response

We now explicitly point to that in the discussion (lines 560-562) and added a reference to that in the introduction: <<In the Discussion section, we return to this and its importance for designing tailored communication so as to avoid fallacious inferences.>>

Comment block 3

- At several points it seems like the main research gap is about the lack of study on deference behavior, but you never define deference and it's not emphasized as a main question in the title, abstract, or literature review. If it is a main idea, it needs to be defined and reviewed. In other places, the emphasis is on heuristics, but they are likewise not fully introduced.

Response

The focus is on how people navigate the complex landscapes of evidence to make decisions, including the role of deference behavior, and use heuristics. We have added explanations of concept mentioned in the abstract and the introduction (including “deference” and “heuristics” among others).

Comment block 4

- "Expert source" is never defined in the context of your work

- Similarly, line 129 introduces the idea of cognitive reflection, a complex topic. It is never defined or reviewed and you do not explain the relationship between cognitive reflection and heuristic reasoning.

- In general, the theoretical background is very weak. All terms need definitions and important concepts need more space in the review, especially since these topics are very complex and their definitions have sometimes been unclear in prior literature.

- It would help to add subsections to the introduction to introduce the reader to the different ideas and organize your aims and predictions in a "current study" section.

Response

In general, we largely expanded the introduction to include definitions and review of important concepts. In particular, we have added the definition of “Expert source” and reviewed it, in relation to deference. (lines 58-71) and defined ‘‘cognitive reflection’’ (lines 137-138). We also added subsections to the introduction to organize the research gap and our contributions.

Comment block 5

Methods, Results:

- The data being a convenience sample collected online over a single day is of course a limitation. You mention that it is a representative sample, but in supplement section 1.1 it seems that this claim is based on the numbers just being similar to census data, without a formal analysis. There are only two gender categories and three political affiliation categories, which, although is probably based on the restrictions of the census data, is also a limitation.

Response

We removed “representative sample” from the Appendix where it appeared. We have also discussed the limitations of voluntary response sampling (lines 613-615). In regard to gender, our questionnaire offered the options: “Male”, “Female”, “Non-binary / third gender”, and “Prefer not to say”.

Comment block 6

- You did not find effects of political ideology, so this idea does not need to be emphasized, but you do need to briefly introduce why you might think there may be an effect (or a confounding effect) based on previous research.

Response

We added a paragraph on “political ideology” in the Methods section (lines 300-307). We explain why one might in general expect an effect based on previous research, but also how our specific design choices were made to minimize any confounding effect of political ideology.

Comment block 7

- Where did the items in supplement table II come from? Was there any piloting of the items?

Response

There was no piloting of the items. Items ev1-ev4 are simply the content of a 2 x 2 contingency table, with number chosen so that the use of heuristic processing is likely to result in a wrong conclusion. Items ev5-ev12 were selected to represent a diversity of evidence different from the 2 x 2 contingency table, appealing to some authoritative source (manufacturer, scientists, doctors, …). The items have been moved to the Methods in the main text (Table 2).

Comment block 8

-ev1-ev4 are very similar and at least conceptually complementary pairwise (we don't know how many total employees the company has, so maybe the math doesn't add up), whereas the rest of the items are more independent from each other. How does this affect the participant behavior and the analysis? You mention that some of those items are correlated (line 276) so it seems intentional. It is also unclear how the base rate neglect and confounders neglect heuristics apply to these 4 evidence items vs the rest of the items in terms of how respondents use the information, especially when they can control how many pieces of evidence they are exposed to.

Response

The total number of employees is the sum of the numbers in ev1-ev4, and we specified this at line 172 in the sentence: <<Respondents were informed that “a few months ago, a major midwestern company offered this new nasal spray for free to all of its employees. Last week, the firm conducted a survey of its employees. In this poll, people were asked if they used the nasal spray as directed and if they have since gotten sick with COVID-19.”>>

The pieces of evidence ev1-ev4 form together the full “statistical” type of evidence. They are indeed correlated. One of our objectives was to offer more fine-grained evidence and observe how people select from these pieces of evidence while keeping the poss

Attachment Submitted filename: Response to Reviewers.pdf

# Peer review: Decision Letter 1

**DOI:** 10.1371/journal.pone.0352096.r003

**Authors:** Fernando Blanco

19 May 2026

PONE-D-25-61437R1

Patterns between evidence seeking behaviors, reasoning, and cognitive reflection: a supervised clustering approach

PLOS One

Dear Dr. Tovissodé,

Thank you for submitting your manuscript to PLOS ONE. After careful consideration, we feel that it has merit but does not fully meet PLOS ONE’s publication criteria as it currently stands. Therefore, we invite you to submit a revised version of the manuscript that addresses the points raised during the review process.

EDITOR'S COMMENT: I would like to reiterate my apologies for the lengthy review process. However, I am satisfied with the quality and expertise of the two reviewers who have worked on this review, and I would like to thank them for their dedication.

The revisions in this version of the article are both positive. I agree with Reviewer 2’s comment regarding the concept of ‘deference’, so my decision is to accept the article with the condition that a definition and a more detailed explanation are provided, as suggested by this reviewer. Additionally, the inclusion of the reflection test results and predictions in the abstract, as suggested by Reviewer 1, would be a good idea.

In any case, I agree with both reviewrs in that the article has improved substantially and is almost ready for acceptance. I do not think that I will need to send for review the next revision of the paper.

Please submit your revised manuscript by Jul 03 2026 11:59PM. If you will need more time than this to complete your revisions, please reply to this message or contact the journal office at plosone@plos.org. When you're ready to submit your revision, log on to https://www.editorialmanager.com/pone/ and select the 'Submissions Needing Revision' folder to locate your manuscript file.

Please include the following items when submitting your revised manuscript:

- A letter that responds to each point raised by the academic editor and reviewer(s). You should upload this letter as a separate file labeled 'Response to Reviewers'.
- A marked-up copy of your manuscript that highlights changes made to the original version. You should upload this as a separate file labeled 'Revised Manuscript with Track Changes'.
- An unmarked version of your revised paper without tracked changes. You should upload this as a separate file labeled 'Manuscript'.

If you would like to make changes to your financial disclosure, please include your updated statement in your cover letter. Guidelines for resubmitting your figure files are available below the reviewer comments at the end of this letter.

If applicable, we recommend that you deposit your laboratory protocols in protocols.io to enhance the reproducibility of your results. Protocols.io assigns your protocol its own identifier (DOI) so that it can be cited independently in the future. For instructions see: https://journals.plos.org/plosone/s/submission-guidelines#loc-laboratory-protocols. Additionally, PLOS ONE offers an option for publishing peer-reviewed Lab Protocol articles, which describe protocols hosted on protocols.io. Read more information on sharing protocols at https://plos.org/protocols?utm_medium=editorial-email&utm_source=authorletters&utm_campaign=protocols.

As the corresponding author, your ORCID iD is verified in the submission system and will appear in the published article. PLOS supports the use of ORCID, and we encourage all coauthors to register for an ORCID iD and use it as well. Please encourage your coauthors to verify their ORCID iD within the submission system before final acceptance, as unverified ORCID iDs will not appear in the published article. *Only*  the individual author can complete the verification step; PLOS staff *cannot*  verify ORCID iDs on behalf of authors.

We look forward to receiving your revised manuscript.

Kind regards,

Fernando Blanco

Academic Editor

PLOS One

Journal Requirements:

If the reviewer comments include a recommendation to cite specific previously published works, please review and evaluate these publications to determine whether they are relevant and should be cited. There is no requirement to cite these works unless the editor has indicated otherwise.

Please review your reference list to ensure that it is complete and correct. If you have cited papers that have been retracted, please include the rationale for doing so in the manuscript text, or remove these references and replace them with relevant current references. Any changes to the reference list should be mentioned in the rebuttal letter that accompanies your revised manuscript. If you need to cite a retracted article, indicate the article’s retracted status in the References list and also include a citation and full reference for the retraction notice.

Additional Editor Comments:

Firstly, I would like to reiterate my apologies for the lengthy review process. However, I am satisfied with the quality and expertise of the two reviewers who have worked on this review, and I would like to thank them for their dedication.

The revisions in this version of the article are both positive. I agree with Reviewer 2’s comment regarding the concept of ‘deference’, so my decision is to accept the article with the condition that a definition and a more detailed explanation are provided, as suggested by this reviewer. I consider that the article has improved substantially and is almost ready for acceptance.

[Note: HTML markup is below. Please do not edit.]

Reviewers' comments:

Reviewer's Responses to Questions

**Comments to the Author**

1. If the authors have adequately addressed your comments raised in a previous round of review and you feel that this manuscript is now acceptable for publication, you may indicate that here to bypass the “Comments to the Author” section, enter your conflict of interest statement in the “Confidential to Editor” section, and submit your "Accept" recommendation.

Reviewer #1: All comments have been addressed

Reviewer #2: (No Response)

**********

2. Is the manuscript technically sound, and do the data support the conclusions?

The manuscript must describe a technically sound piece of scientific research with data that supports the conclusions. Experiments must have been conducted rigorously, with appropriate controls, replication, and sample sizes. The conclusions must be drawn appropriately based on the data presented.

Reviewer #1: Yes

Reviewer #2: Yes

**********

3. Has the statistical analysis been performed appropriately and rigorously?

Reviewer #1: Yes

Reviewer #2: Yes

**********

4. Have the authors made all data underlying the findings in their manuscript fully available?

The PLOS Data policy requires authors to make all data underlying the findings described in their manuscript fully available without restriction, with rare exception (please refer to the Data Availability Statement in the manuscript PDF file). The data should be provided as part of the manuscript or its supporting information, or deposited to a public repository. For example, in addition to summary statistics, the data points behind means, medians and variance measures should be available. If there are restrictions on publicly sharing data—e.g. participant privacy or use of data from a third party—those must be specified.

Reviewer #1: Yes

Reviewer #2: Yes

**********

5. Is the manuscript presented in an intelligible fashion and written in standard English?

PLOS ONE does not copyedit accepted manuscripts, so the language in submitted articles must be clear, correct, and unambiguous. Any typographical or grammatical errors should be corrected at revision, so please note any specific errors here.

Reviewer #1: Yes

Reviewer #2: Yes

**********

6. Review Comments to the Author

Please use the space provided to explain your answers to the questions above. You may also include additional comments for the author, including concerns about dual publication, research ethics, or publication ethics. (Please upload your review as an attachment if it exceeds 20,000 characters)

Reviewer #1: I found the revisions satisfactory and, thus, can recommend them for publication.

I also appreciated some of the improvements that Reviewer 2 elicited. But, for whatever this is worth, I found some of the authors' replies to Reviewer 2 to be supererogatory. For example, 'folk' is standard terminology in some of my fields (e.g., experimental philosophy) and, thus, I found no need for Reviewer 2's comment about it (or the author's compliant removal of the term). So even if Reviewer 2 were insufficiently satisfied with the revisions, I would not consider that — by itself — a reason to reject the revised manuscript. I think the authors did more than their duty.

If, however, the editor were to request further revision, I think one very minor opportunity to improve the paper would involve explicit integration of the reflection test result(s) into the paper's abstract. As far as I can tell, the fact that reflection test performance predicts less neglect of relevant evidence provides some independent evidence of some of the authors' claims. So without explicit mention of what reflection test performance predicts, the abstract may not relay as much of the paper's supporting evidence as it could.

In case itemized responses are beneficial for the final decision, I include them below:

Re: My Comment 1

The authors adequately integrated the explication of 'reflection' and the psychometric investigations of its tests.

Re: My Comment 2

The authors explained that I may have been looking at the wrong table in the ancillary materials (V instead of IV). I seem to have been confused by the cross-reference in this sentence: "unsupervised clustering actually results in LESS well-separated... clusters (Table V)." The new Supplement seems to confirm what the authors claim in the paper (and explain in their replies): "Table II displays cluster validation measures for supervised and unsupervised hierarchical clustering methods. It appears that supervised clustering provides the most well separated (higher silhouette score) and most stable clusters (lower APN) for all considered k values."

Re: My Comment 3

The authors added something like the simplest valid analysis that I proposed. (I think this is what is now reported in Table VI.)

Re: My Comment 4

My comments about typos and minor issues also seem to have been resolved.

Nick Byrd (I sign all of my reviews)

Reviewer #2: I thank the authors for addressing my comments and commend them for improving their paper immensely. I have only a minor revision request at this point.

The abstract is now much more clear and the goals of the paper are clearer as well. However, the concept of "deference" still comes into the abstract at the very end, with no mention of what it is or why it is involved with the main results. Additionally, the sentence "Consequently, evidential categories in information seeking research are likely overly monolithic, despite their advantage of studying a wider range of evidential categories used in decision making" is difficult to understand.

The introduction is much improved! The new structure greatly helps clarify your position and goals and addressed most of my comments.

I would still need more of an actual definition for "deference"--line 70; if it is an important construct in the paper, it should be defined explicitly, not obliquely. I appreciate that the distinctions between "first-order" and "second-order" evidence are mixed in the literature. But it is also still a bit unclear to me how "deference" differs from theories of epistemic justifications as you've mentioned briefly, e.g. justification by authority (for example, Meng-Jung Tsai, Ching-Yeh Wang, An-Hsuan Wu, Ivar Bråten, Differences in epistemic justification profiles during conflicting socio-scientific information processing: A study of visual and memory-based behavior via eye-tracking, Acta Psychologica, Volume 252, 2025, https://doi.org/10.1016/j.actpsy.2024.104680. (https://www.sciencedirect.com/science/article/pii/S0001691824005584). And additionally it is unclear where the construct comes from. Is it a novel idea meant as an umbrella for several related constructs? Or something coming from previous literature? You mention that you use deference to avoid making assumptions about who participants would believe are "experts" (and thus, authorities?). The ideas are almost there in the discussion around lines 58-79, but it is still unclear how deference relates to the existing concept of a "source feature" of evidence, which as I understand it, would be related to second-order evidence. It is very important to be clear about your claims about this construct, since your analytical approach uses weights on information *sources*. Deference would then be directly related to the "source features" as participants understand them, e.g. authority, trustworthiness, expertise, etc.

Overall, this manuscript has been greatly improved. I only have the last comments about the importance of the "deference" construct in relation to the authors' claims and use of it and its relationship to existing similar constructs. If the authors would address this issue, then I would find the paper suitable to publish.

**********

7. PLOS authors have the option to publish the peer review history of their article (what does this mean?). If published, this will include your full peer review and any attached files.

If you choose “no”, your identity will remain anonymous but your review may still be made public.

**Do you want your identity to be public for this peer review?**  For information about this choice, including consent withdrawal, please see our Privacy Policy.

Reviewer #1: **Yes:** Nick Byrd

Reviewer #2: No

**********

[NOTE: If reviewer comments were submitted as an attachment file, they will be attached to this email and accessible via the submission site. Please log into your account, locate the manuscript record, and check for the action link "View Attachments". If this link does not appear, there are no attachment files.]

To ensure your figures meet our technical requirements, please review our figure guidelines: https://journals.plos.org/plosone/s/figures

You may also use PLOS’s free figure tool, NAAS, to help you prepare publication quality figures: https://journals.plos.org/plosone/s/figures#loc-tools-for-figure-preparation.

NAAS will assess whether your figures meet our technical requirements by comparing each figure against our figure specifications.

# Author comment: Author response to Decision Letter 2

**DOI:** 10.1371/journal.pone.0352096.r004

3 Jun 2026

Dear Editor of Plos One,

I am submitting this letter in response to the decision: Revision required [PONE-D-25-61437R1] (by July 03, 2026).

Thank you for considering our revisions and giving us the opportunity to improve our manuscript. We are grateful to the two reviewers for their comments and constructive suggestions to improve the abstract and introduction. We provide a point-by-point answer to reviewers’ comments in the following pages. The reviewers’ comments are indicated by the color blue, and the responses to comments are indicated by the color red.

Best regards,

For the authors,

Chenangnon Tovissode

Reviewer #1

I found the revisions satisfactory and, thus, can recommend them for publication. I also appreciated some of the improvements that Reviewer 2 elicited.

Comment. If, however, the editor were to request further revision, I think one very minor opportunity to improve the paper would involve explicit integration of the reflection test result(s) into the paper's abstract. As far as I can tell, the fact that reflection test performance predicts less neglect of relevant evidence provides some independent evidence of some of the authors' claims. So without explicit mention of what reflection test performance predicts, the abstract may not relay as much of the paper's supporting evidence as it could.

Response

We thank the reviewer for pointing out the lack of this important result in the abstract. We have explicitly included the reflection test results as independent evidence supporting our conclusion, by adding the following phrase: <<... we find that higher performance on the cognitive reflection test predicts selection of the full data of a 2 x 2 contingency table.>>

Reviewer #2

I thank the authors for addressing my comments and commend them for improving their paper immensely. I have only a minor revision request at this point.

The abstract is now much more clear and the goals of the paper are clearer as well.

Comment block 1

However, the concept of "deference" still comes into the abstract at the very end, with no mention of what it is or why it is involved with the main results. Additionally, the sentence "Consequently, evidential categories in information seeking research are likely overly monolithic, despite their advantage of studying a wider range of evidential categories used in decision making" is difficult to understand.

Response

We have revised the abstract to include these two sentences which provide an inline definition of deference and clarify the unclear sentence: <<But, the latter group is nevertheless similar to base rate neglecters in who they consider to be relevant outside sources (i.e., they have roughly the same ``deference'' behavior). So while evidential categories in information seeking research are well suited to track differences in deference behaviors, they are blind to the difference between these two groups.>> This also clarifies why "deference" is involved in the main results.

Comment block 2

The introduction is much improved! The new structure greatly helps clarify your position and goals and addressed most of my comments.

I would still need more of an actual definition for "deference"line 70; if it is an important construct in the paper, it should be defined explicitly, not obliquely. I appreciate that the distinctions between "first-order" and "second-order" evidence are mixed in the literature. But it is also still a bit unclear to me how "deference" differs from theories of epistemic justifications as you've mentioned briefly, e.g. justification by authority (for example, Meng-Jung Tsai, Ching-Yeh Wang, An-Hsuan Wu, Ivar Bråten, Differences in epistemic justification profiles during conflicting socio-scientific information processing: A study of visual and memory-based behavior via eye-tracking, Acta Psychologica, Volume 252, 2025, https://doi.org/10.1016/j.actpsy.2024.104680. (https://www.sciencedirect.com/science/article/pii/S0001691824005584). And additionally it is unclear where the construct comes from. Is it a novel idea meant as an umbrella for several related constructs? Or something coming from previous literature? You mention that you use deference to avoid making assumptions about who participants would believe are "experts" (and thus, authorities?).

The ideas are almost there in the discussion around lines 58-79, but it is still unclear how deference relates to the existing concept of a "source feature" of evidence, which as I understand it, would be related to second-order evidence. It is very important to be clear about your claims about this construct, since your analytical approach uses weights on information *sources*. Deference would then be directly related to the "source features" as participants understand them, e.g. authority, trustworthiness, expertise, etc.

Overall, this manuscript has been greatly improved. I only have the last comments about the importance of the "deference" construct in relation to the authors' claims and use of it and its relationship to existing similar constructs. If the authors would address this issue, then I would find the paper suitable to publish.

Response

We greatly appreciate this comment which prompted us to clarify the term "deference" in the paper. The concept of deference in this work is not a construct; hence we do not make a comparison with existing theories of epistemic justifications. It is not a novel idea meant as an umbrella for several related constructs. By deference, we simply mean consideration of pre-processed information as relevant. Here is the complete revised paragraph where we introduce the term in the introduction:

<< What we would like to do is offer people, in a controlled setting, a wider and more fine-grained selection of evidence that spans both gathering and processing research. For the processing side, we will use “statistical” to mean the entries in a contingency table (but unprocessed, so to speak). This enables us to detect the possibilities of processing heuristics. On the information gathering side we will use “deference” to mean sources where information has been pre-processed or given an interpretation (e.g. a recommendation) and seen as potentially relevant without the presumption that subjects see the sources as experts. Now if we give subjects even just 12 options to select from across these two sides, we face the problem of combinatorial explosion that the typologies of evidence are meant to make tractable, especially if we consider the full pathway of gathering information and its processing (e.g. by making a judgment about causal efficacy). In the next section we illustrate an analytical approach for handling this challenge. >>

This is indeed related to second-order evidence, and to many concepts in the literature, as we discussed when comparing our empirical results to existing theoretical delineations of evidence categories.

Attachment Submitted filename: Response_to_Reviewers.pdf

# Editor report: Decision Letter 2

**DOI:** 10.1371/journal.pone.0352096.r005

**Authors:** Fernando Blanco

4 Jun 2026

Patterns between evidence seeking behaviors, reasoning, and cognitive reflection: a supervised clustering approach

PONE-D-25-61437R2

Dear Dr. Tovissodé,

We’re pleased to inform you that your manuscript has been judged scientifically suitable for publication and will be formally accepted for publication once it meets all outstanding technical requirements.

Within one week, you’ll receive an e-mail detailing the required amendments. When these have been addressed, you’ll receive a formal acceptance letter and your manuscript will be scheduled for publication.

An invoice will be generated when your article is formally accepted. Please note, if your institution has a publishing partnership with PLOS and your article meets the relevant criteria, all or part of your publication costs will be covered. Please make sure your user information is up-to-date by logging into Editorial Manager at Editorial Manager® and clicking the ‘Update My Information' link at the top of the page. For questions related to billing, please contact billing support.

If your institution or institutions have a press office, please notify them about your upcoming paper to help maximize its impact. If they’ll be preparing press materials, please inform our press team as soon as possible -- no later than 48 hours after receiving the formal acceptance. Your manuscript will remain under strict press embargo until 2 pm Eastern Time on the date of publication. For more information, please contact onepress@plos.org.

Kind regards,

Fernando Blanco

Academic Editor

PLOS One

Additional Editor Comments (optional):

Reviewers' comments:

# Editor report: Acceptance letter

**DOI:** 10.1371/journal.pone.0352096.r006

**Authors:** Fernando Blanco

PONE-D-25-61437R2

PLOS One

Dear Dr. Tovissodé,

I'm pleased to inform you that your manuscript has been deemed suitable for publication in PLOS One. Congratulations! Your manuscript is now being handed over to our production team.

At this stage, our production department will prepare your paper for publication. This includes ensuring the following:

* All references, tables, and figures are properly cited

* All relevant supporting information is included in the manuscript submission,

* There are no issues that prevent the paper from being properly typeset

You will receive further instructions from the production team, including instructions on how to review your proof when it is ready. Please keep in mind that we are working through a large volume of accepted articles, so please give us a few days to review your paper and let you know the next and final steps.

Lastly, if your institution or institutions have a press office, please let them know about your upcoming paper now to help maximize its impact. If they'll be preparing press materials, please inform our press team within the next 48 hours. Your manuscript will remain under strict press embargo until 2 pm Eastern Time on the date of publication. For more information, please contact onepress@plos.org.

You will receive an invoice from PLOS for your publication fee after your manuscript has reached the completed accept phase. If you receive an email requesting payment before acceptance or for any other service, this may be a phishing scheme. Learn how to identify phishing emails and protect your accounts at https://explore.plos.org/phishing.

If we can help with anything else, please email us at customercare@plos.org.

Thank you for submitting your work to PLOS ONE and supporting open access.

Kind regards,

PLOS ONE Editorial Office Staff

on behalf of

Dr. Fernando Blanco

Academic Editor

PLOS One
